# Calcium Handling Defects and Cardiac Arrhythmia Syndromes

**DOI:** 10.3389/fphar.2020.00072

**Published:** 2020-02-25

**Authors:** Kornél Kistamás, Roland Veress, Balázs Horváth, Tamás Bányász, Péter P. Nánási, David A. Eisner

**Affiliations:** ^1^Department of Physiology, Faculty of Medicine, University of Debrecen, Debrecen, Hungary; ^2^Division of Cardiovascular Sciences, School of Medical Sciences, University of Manchester, Manchester, United Kingdom; ^3^Department of Dental Physiology, Faculty of Dentistry, University of Debrecen, Debrecen, Hungary

**Keywords:** calcium signalling, cardiac arrhythmias, catecholaminergic polymorphic ventricular tachycardia, long QT syndrome, atrial fibrillation, reentry, early afterdepolarization, delayed afterdepolarization

## Abstract

Calcium ions (Ca^2+^) play a major role in the cardiac excitation-contraction coupling. Intracellular Ca^2+^ concentration increases during systole and falls in diastole thereby determining cardiac contraction and relaxation. Normal cardiac function also requires perfect organization of the ion currents at the cellular level to drive action potentials and to maintain action potential propagation and electrical homogeneity at the tissue level. Any imbalance in Ca^2+^ homeostasis of a cardiac myocyte can lead to electrical disturbances. This review aims to discuss cardiac physiology and pathophysiology from the elementary membrane processes that can cause the electrical instability of the ventricular myocytes through intracellular Ca^2+^ handling maladies to inherited and acquired arrhythmias. Finally, the paper will discuss the current therapeutic approaches targeting cardiac arrhythmias.

## Introduction

**Figure 1:**
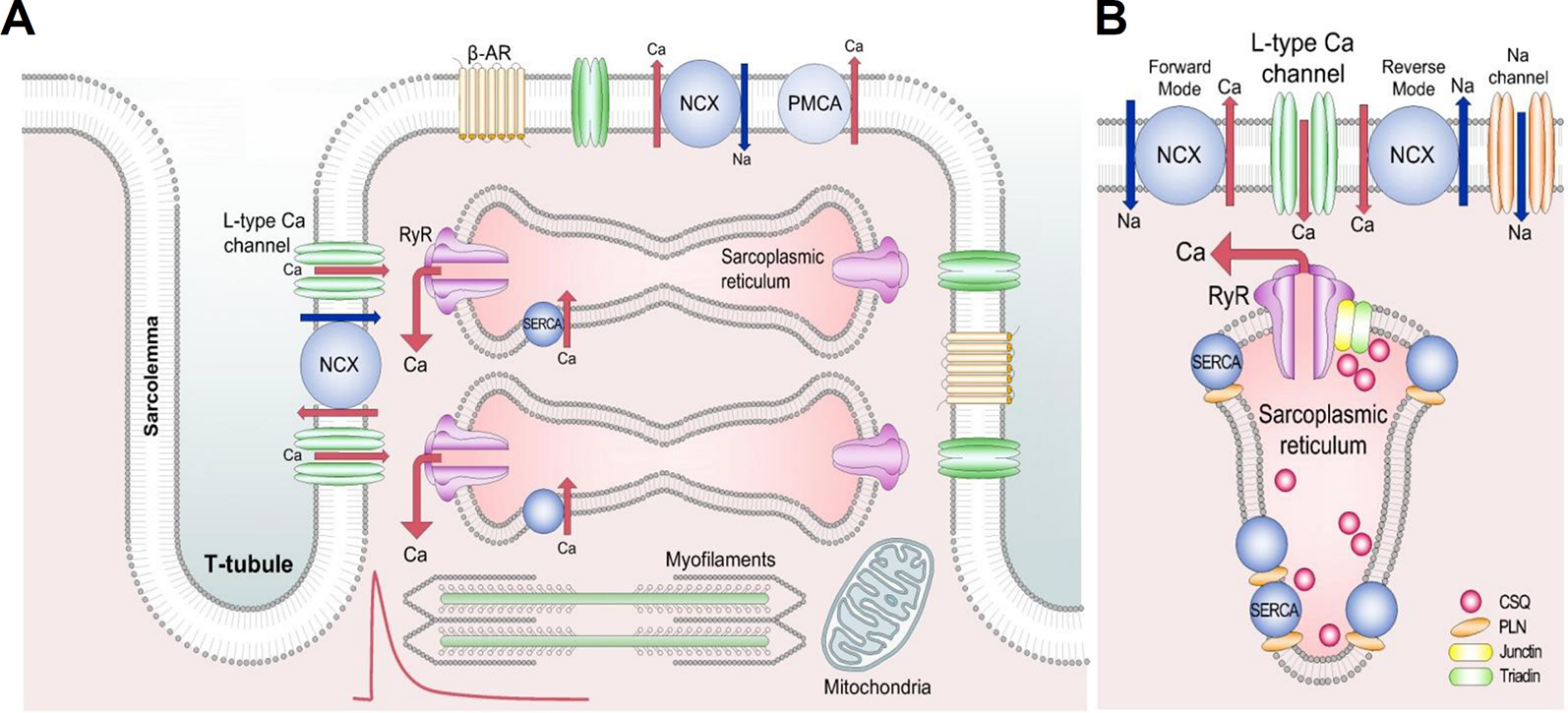
Schematic diagram of the cardiac excitation-contraction coupling. **(A)** Structures involved in Ca^2+^ transport in cardiac mycocytes. Red trace shows a typical systolic Ca^2+^ transient. Briefly, during the Ca^2+^-induced Ca^2+^ release process, Ca^2+^ entering the cell *via* L-type Ca^2+^ channels releases a larger amount of Ca^2+^ from the sarcoplasmic reticulum to activate the contractile machinery. Ca^2+^ extrusion requires NCX, PMCA, and SERCA. **(B)** Detailed section of the dyad showing the major proteins involved in Ca^2+^ cycling. Reproduced from Eisner et al. used with permission (Eisner et al., 2017). β-AR, β adrenoceptor; NCX, Na^+^-Ca^2+^ exchange; PMCA, plasma membrane Ca^2+^-ATPase; RyR, ryanodine receptor; SERCA, sarco/endoplasmic reticulum Ca^2+^-ATPase; CSQ, calsequestrin; PLN, phospholamban.

Excitation-contraction coupling (E-C coupling) of the cardiac myocytes is a well studied phenomenon. We know that the calcium ion (Ca^2+^) plays a major role in controlling contraction and force, a feature that was originally described by Sidney Ringer more than a century ago (Ringer, 1883). Since this discovery, it has become clear that changes in intracellular Ca^2+^ concentration ([Ca^2+^]_i_) have a significant role in virtually all parts of the human body. Of particular importance is the fact, that within cardiac myocytes, [Ca^2+^]_i_ changes must be tightly regulated, so that the heart can beat rhythmically. This means that during the cardiac systole, [Ca^2+^]_i_ has to increase to certain levels to make contraction occur and must fall in diastole, allowing the muscle to relax and prepare for the next cardiac cycle. E-C coupling has been reviewed in detail (Bers, 2002; Eisner et al., 2017), here we consider the elementary steps and the events that can lead to electrical disturbances (**Figure 1**).

The normal cardiac action potential (AP) originates in the sinoatrial node and propagates through the heart. In the ventricle the initial depolarization opens voltage-gated sodium channels leading to further depolarization which, in turn, opens the L-type Ca^2+^ channels, causing a large Ca^2+^-influx (**Figure 1A**). Some Ca^2+^ can also enter *via* T-type Ca^2+^ channels and reverse mode Na^+^/Ca^2+^ exchange (NCX) (Kohomoto et al., 1994; Sipido et al., 1997). This Ca^2+^ entry triggers a process known as calcium-induced calcium release (CICR), in which Ca^2+^ is released from the sarcoplasmic reticulum (SR) into the cytoplasm *via* ryanodine receptors (RyR), allowing Ca^2+^ to bind to the myofilament protein troponin C, activating the contractile machinery. Normal cardiac function also requires relaxation to occur; this results from a decrease of free cytoplasmic Ca^2+^ levels. Several Ca^2+^ transport pathways are involved in this process, as Ca^2+^ reuptake into the SR by the SR Ca^2+^-ATPase (SERCA), Ca^2+^ extrusion by the sarcolemmal NCX and plasma membrane Ca^2+^-ATPase (PMCA) (**Figure 1B**) (Bers, 2000). This normal cardiac function requires perfect coordination of the ion currents and intracellular processes, as any imbalance in Ca^2+^ homeostasis of a cardiac myocyte can lead to electrical disturbances (from cellular AP prolongation to complex arrhythmic storms) (Eisner et al., 2017; Eisner, 2018).

Here we review the role of Ca^2+^ in generating and maintaining cardiac arrhythmias from basic arrhythmia mechanisms to recent progresses in pharmacological challenges and possible future therapies.

## Calcium in Pathophysiology, Arrhythmia Mechanisms

**Figure 2:**
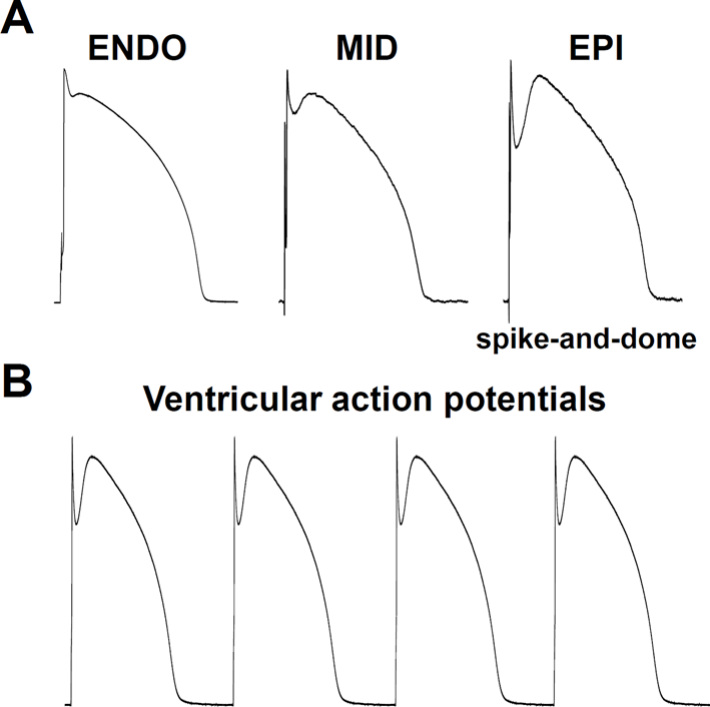
Cellular physiological electrical activities. **(A)** Transmural heterogeneity in the cardiac ventricular action potential, showing (from left to right) recordings from: subendocardium, midmyocardium, and subepicardium. Note the spike-and-dome action potential configuration in the subepicardium. ENDO, subendocardial mycocyte; MID, midmyocardial “M” myocyte; EPI, subepicardial myocyte. **(B)** Series of typical subepicardial ventricular action potentials at normal pacing activity.

**Figure 3:**
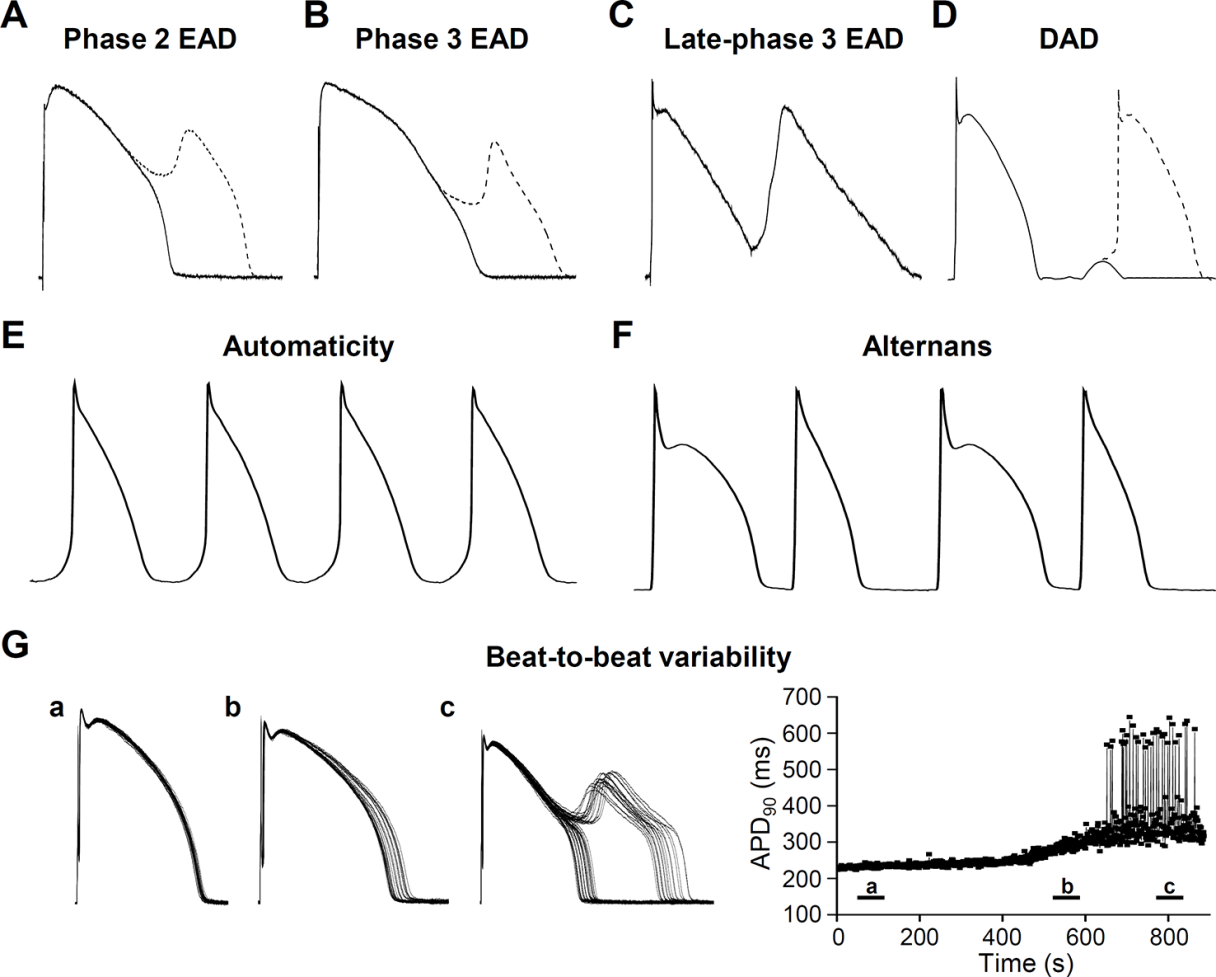
Cellular pathophysiological electrical activities. **(A)** Phase 2 early afterdepolarization (EAD), **(B)** Phase 3 EAD, **(C)** Late-phase 3 EAD, **(D)** Delayed afterdepolarization (DAD) manifesting triggered activity. Ca^2+^ has an important role in generating afterdepolarizations. Underlying mechanisms are described in the relevant sections. **(E)** Automaticity (spontaneous membrane potential oscillations) occurs if the membrane potential of the cells shift to more positive values causing abnormal activity. **(F)** Cardiac voltage alternans, manifesting a long-short-long-short pattern. **(G)** Short term beat-to-beat variability of the action potential duration. **(a)**, **(b)**, and **(c)** show different time points after interventions that increase action potential duration and beat-to-beat variability leading to EAD generation. Right panel of **(G)** shows action potential duration at 90% of the repolarization (APD_90_) as a function of time.

Arrhythmia mechanisms have multiscale dynamics in the heart. The lower end is the molecular scale, originating from the stochastic behavior of ion channels, resulting from thermodynamic fluctuations (Qu and Weiss, 2015). Next is the cellular scale, with differences in the shape of the APs originating from distant parts of the myocardium (**Figure 2A**). Under some diseased conditions, several mechanisms can lead to electrical disturbances at the cellular level, including early or delayed afterdepolarizations (EAD or DAD, respectively) (**Figures 3A–D**). Whole-cell Ca^2+^ oscillations, developing into propagating Ca^2+^ waves arise when the molecular and cellular dynamics merge at the tissue and organ level. The lower and higher scales tend to have a bidirectional information flow. A good example is when EADs arising during an AP due to abnormal ion currents and Ca^2+^ dynamics, can bring an extra amount of Ca^2+^ into the cell due to L-type Ca^2+^ channel reopening and potentiate Ca^2+^ waves. These multiscale dynamics can lead to life threatening complex arrhythmias.

**Figure 4:**
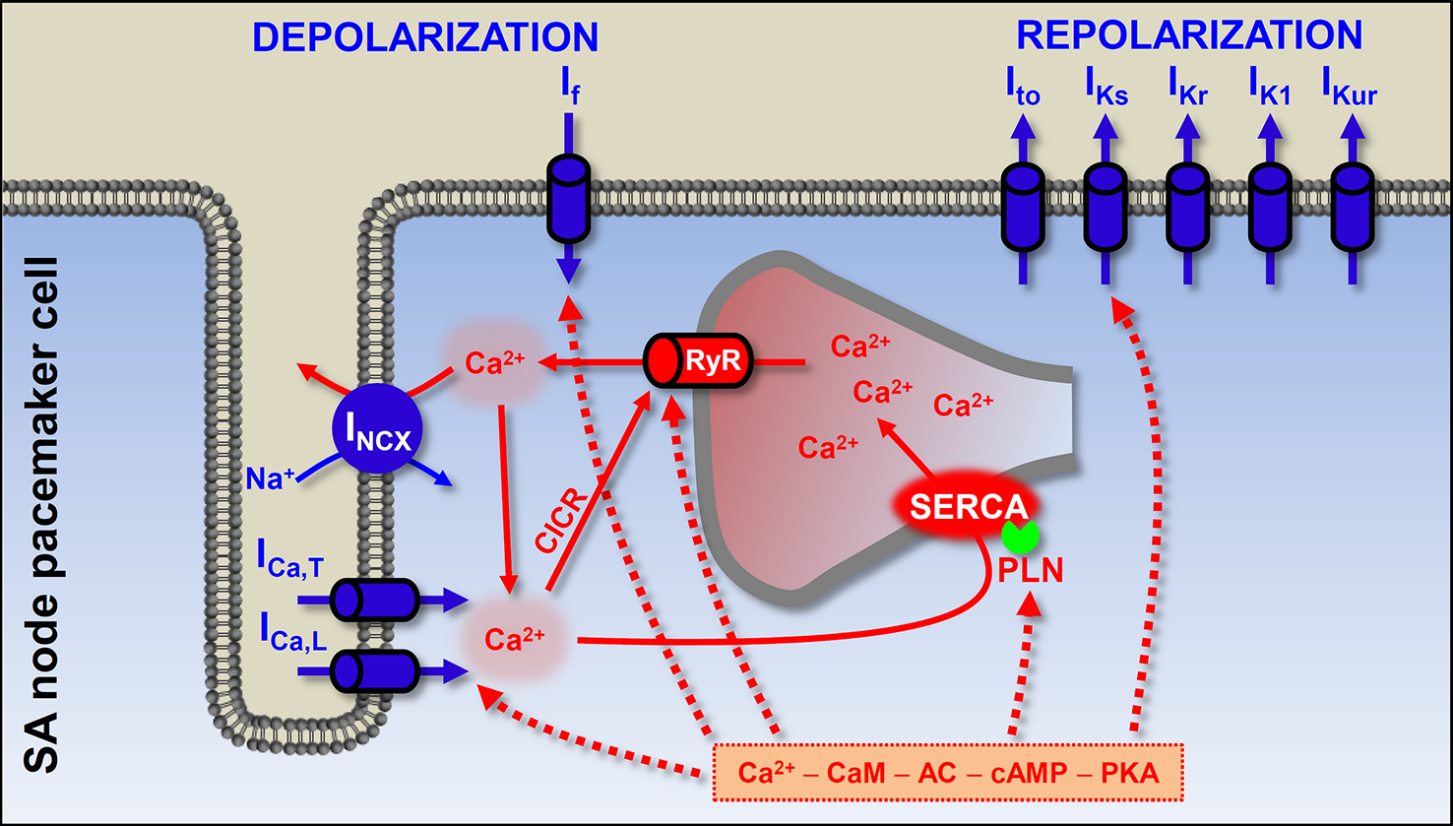
The origin of the heartbeat: coupled-clock pacemaker system in the sinoatrial cells. The pacemaker activity of the SA node originates from the membrane and calcium clock mechanisms. The former is composed of the sarcolemmal channel proteins, and the latter results from sarcoplasmic reticulum and sarcoplasmic Ca^2+^ turnover. At end of the SA action potential the hyperpolarization-activated I_f_ depolarizes the membrane to a level where Ca^2+^ channels open. In addition, during late diastole, spontaneous SR Ca^2+^ releases further depolarize the membrane by activating I_NCX_. Ca^2+^ can bind to calmodulin and activate adenylyl cyclase (AC). High constitutive activation of AC leading to high basal level of cAMP (which is needed for protein kinase A-dependent phosphorylation) in SA node cells has been suggested to contribute to the Ca^2+^ overload state. PKA-dependent phosphorylation of phospholamban, I_Ca,L_, and RyR promotes spontaneous Ca^2+^ release. Blue shows the membrane clock and red shows the calcium clock mechanism. Solid arrows show the Ca^2+^-induced Ca^2+^ release process and spontaneous Ca^2+^ release events *via* RyR; dashed arrows show the phosphorylation targets of the cAMP–PKA pathway. I_Ca,L_, L-type Ca^2+^ current; I_Ca,T_, T-type Ca^2+^ current; I_NCX_, Na^+^-Ca^2+^ exchange; I_f_, funny current; I_to_, transient outward K^+^ current; I_Ks_, slow component of delayed rectifier K^+^ current; I_Kr_, rapid component of delayed rectifier K^+^ current; I_K1_, inward rectifier K^+^ current; I_Kur_, ultra rapid component of delayed rectifier K^+^ current; RyR, ryanodine receptor; SERCA, sarcoplasmic reticulum Ca^2+^-ATPase; PLN, phospholamban; CaM, calmodulin; AC, adenylyl cyclase; PKA, protein kinase A; CICR, Ca^2+^-induced Ca^2+^ release; SA, sinoatrial.

Normal cardiac automaticity originates in the sinoatrial (SA) node. If SA node impulse generation is impaired, atrioventricular node (AV node) and Purkinje fibers can show automatic activity. These secondary pacemakers are also called latent or subsidiary pacemakers (Antzelevitch and Burashnikov, 2011). SA node pacemaker activity depends on interactions of membrane potential and [Ca^2+^]_i_. This “coupled-clock” pacemaker system is produced by membrane proteins, driving the AP and the intracellular Ca^2+^ cycling molecules (**Figure 4**) (Maltsev et al., 2006; Lakatta, 2010; Joung et al., 2011).

The “membrane clock” implies sarcolemmal proteins, continuously driving the membrane potential to more positive or more negative values. The most important and well-known participant is the hyperpolarization-activated funny current (I_f_), working mainly during early diastolic depolarization. The consequent depolarization opens Ca^2+^ channels (I_Ca,T_ and I_Ca,L_) and the pacemaker (slow type) action potential occurs. As in the case of the working myocardium, K^+^ currents repolarize the membrane. In the last two decades it has become clear that spontaneous Ca^2+^ release in a cardiac cell is not always pathological. In the “calcium clock” mechanism, spontaneous SR Ca^2+^ release events, the Ca^2+^ sparks activate I_NCX_ and cause late diastolic membrane depolarization. Coupled clock pacemaker system comprises functional interactions between the membrane and calcium clock (**Figure 4**) (Vinogradova et al., 2006; Lakatta and DiFrancesco, 2009; Lakatta et al., 2010).

For physiological contraction and relaxation, not only pacemaker automaticity, but also the impulse conduction system needs to work properly. Spontaneous depolarization from the SA node propagates and depolarizes the distant parts of the cardiac muscle (**Figure 2B**), *via* the SA node, AV node, Bundle of His, Bundle branches, and Purkinje fibers pathway.

Cardiac arrhythmia mechanisms can be divided into two main categories: abnormal impulse formation and abnormal impulse conduction. In general, these arrhythmic events occur when the electrical activity of the heart is slower or faster than normal and/or becomes irregular.

### Abnormal Impulse Generation

Focal activity (enhanced or abnormal impulse generation) is an important arrhythmogenic mechanism and consists of abnormal automaticity and triggered activity.

#### Automaticity

In the normal human heart, the SA node generates the propagating APs and determine the heart rate. In the case of parasystole, when the primary pacemaker is bordered by ischemic, infarcted regions the impulse cannot leave the SA node. Under these conditions, parasystolic pacemakers can take over pacemaker activity and fire APs at a lower rate compared to that of the SA node (Gussak et al., 2003). The AV node produces a junctional rhythm of 40 to 60 bpm and Purkinje fibers of about 20 to 40 bpm (Tse, 2016). In diseased hearts (e.g. heart failure, HF) membrane potential of pacemaker cells can shift to more positive values and this depolarization causes abnormal automaticity. Enhanced activity (i.e. tachycardia) increases rate of AP discharge by three mechanisms: threshold potential shifts to more negative, maximum diastolic potential shifts to more positive, and the rate of phase 4 depolarization increases (**Figure 3E**) (Jalife et al., 2009).

#### Early Afterdepolarization

**Figure 5:**
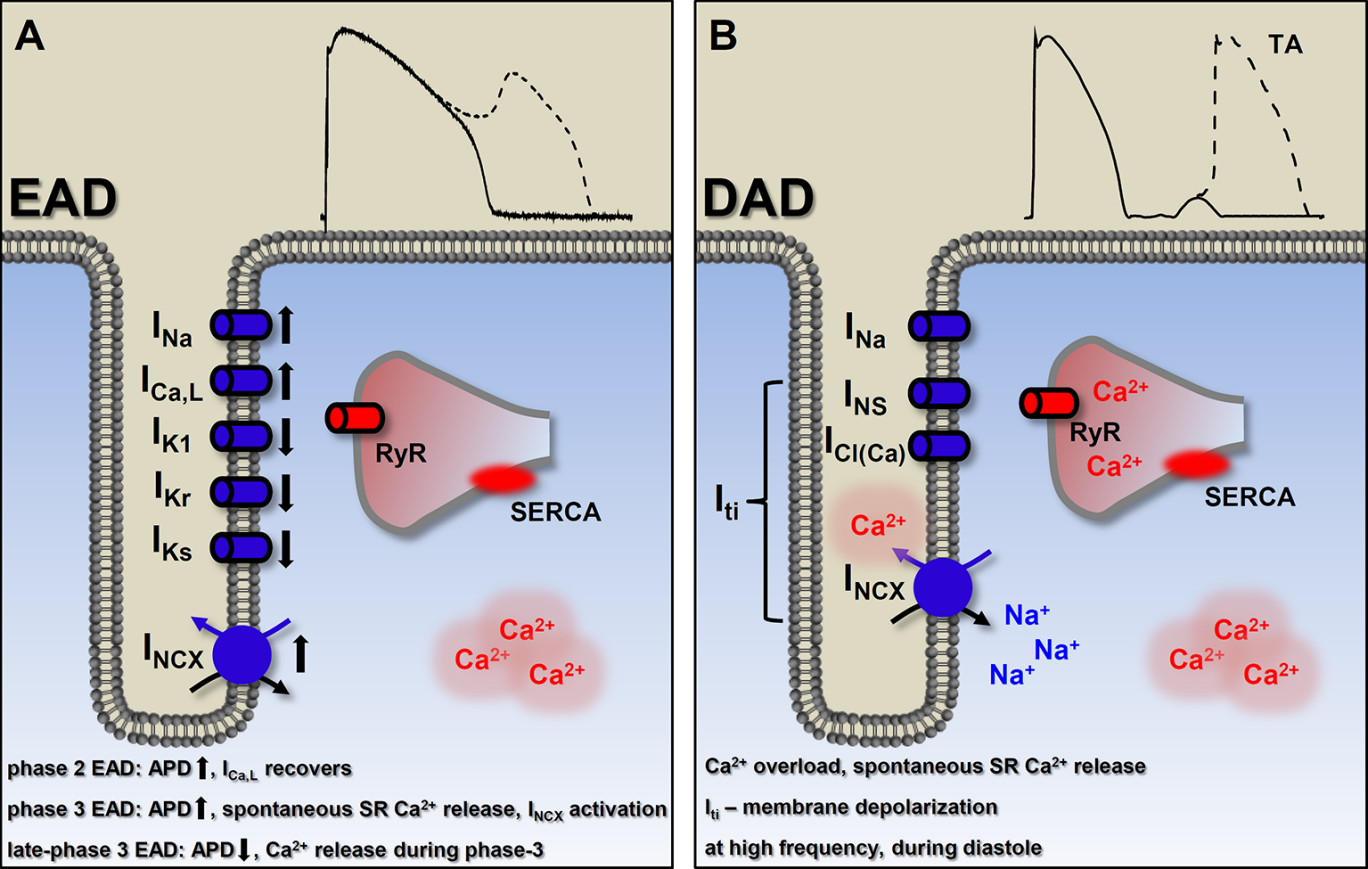
Basic mechanisms of ectopic activity. **(A)** Factors involved in the generation of early afterdepolarizations (EAD). In general, EADs occur when outward currents are reduced (reduced repolarization reserve) and/or the inward currents are enhanced. The currently known types of EADs are consequencies of different etiologies, indicated on **(A)**. Detailed description in the text. Membrane potential recording shows a typical phase 2 EAD. **(B)** Delayed afterdepolarizations (DAD) originate from Ca^2+^ overload and consequently, spontaneous SR Ca^2+^ release which, in turn, generates a depolarizing transient inward (I_ti_) current. Suprathreshold depolarization can elicit triggered activity. Membrane potential recording shows a typical DAD. EAD, early afterdepolarization; DAD, delayed afterdepolarization; I_Ca,L_, L-type Ca^2+^ current; I_Na_, Na^+^ current; I_Ks_, slow component of delayed rectifier K^+^ current; I_Kr_, rapid component of delayed rectifier K^+^ current; I_K1_, inward rectifier K^+^ current; I_NCX_, Na^+^-Ca^2+^ exchange; I_NS_, nonselective Ca^2+^-sensitive cationic currents; I_Cl(Ca)_, Ca^2+^-activated chloride current; I_ti_, transient outward current; RyR, ryanodine receptor; SR, sarcoplasmic reticulum; SERCA, sarcoplasmic reticulum Ca^2+^-ATPase; TA, triggered activity.

Aside from the abnormal automaticity, the most common causes of focal activity are the early and delayed afterdepolarizations (EAD and DAD, respectively). EADs occur before the terminal repolarization (phase 2 and phase 3 repolarization) of the AP, while DADs occur after the repolarization when membrane potential reaches the resting levels (**Figure 5**).

EADs usually occur when repolarization reserve is compromised, i.e. reduced outward currents (I_K1_, I_Kr_, I_Ks_) and/or increased inward currents (I_Na_, window I_Ca,L_, I_NCX_) (Damiano and Rosen, 1984; Sipido et al., 2007; Benitah et al., 2010; Horvath et al., 2015; Karagueuzian et al., 2017), that is, there is a change in the net membrane current during the plateau (**Figure 5A**). In most of the cases these conditions cause prolongation of the AP, allowing I_Ca,L_ to recover from inactivation (Chiamvimonvat et al., 2017) and as a positive feedback loop, triggering an AP (January and Riddle, 1989) (**Figure 3A**). Alternatively, at membrane potentials negative to the activation threshold for I_Ca,L_, spontaneous Ca^2+^ release from the SR can activate I_NCX_, driving a depolarizing current by reactivating I_Na_ (**Figure 3B**) (Szabo et al., 1994). In addition, although EADs usually occur when the AP duration (APD) is prolonged, some data suggests a novel mechanism, where even shortening of APD can be responsible for generation of EADs (late-phase 3 EAD) (Burashnikov and Antzelevitch, 2003). Late-phase 3 EADs occur particularly under elevated intracellular Ca^2+^ loading (i.e. large Ca^2+^ transient) and are considered as a hybrid between EAD and DAD (**Figure 3C**). At normal APD and at membrane potentials negative to the equilibrium of the I_NCX_ (and I_Cl(Ca)_), these Ca^2+^-mediated currents are weakly inward. However, if APD is abbreviated, they become strongly inward, allowing an I_NCX_-driven depolarizing current, when the shorter repolarization allows a stronger (and not spontaneous) Ca^2+^ release from the SR (Burashnikov and Antzelevitch, 2006). The EAD generated under these circumstances interrupts the final phase of the AP. A key difference compared to the previously described EADs (and DADs) is a non-spontaneous Ca^2+^ release in generating late-phase 3 EADs (**Figure 5**). Late-phase 3 EAD also has clinical relevance, as its appearance is immediately following termination of other tachyarrhythmias, such as atrial flutter and fibrillation or ventricular tachycardia and fibrillation (Burashnikov and Antzelevitch, 2006).

The contribution of spontaneous SR Ca^2+^ release and an inward I_NCX_ to the generation of EADs has been described (Priori and Corr, 1990; Volders et al., 1997), furthermore, Volders et al. elegantly demonstrated in isoproterenol induced canine ventricular myocytes that early Ca^2+^ aftertransients and their aftercontractions precede the upstroke of the subsequent EAD so that they are a primary event inducing EADs (Volders et al., 1997). The time course of the EAD generation is characterized by a conditional phase (in other words, an initial delay in repolarization, defined by net membrane current) and the EAD upstroke. In this regard, a significant role of I_NCX_ has been suggested in the initial delay in repolarization, thus in the conditional phase (Volders et al., 2000).

In previous studies, distinct spatial features of afterdepolarization-associated Ca^2+^ transients had been shown; i.e. a heterogenous pattern indicating focal, spontaneous SR Ca^2+^ release in DADs and a homogenous pattern suggesting I_Ca,L_-induced Ca^2+^ release in EADs (Miura et al., 1993; Miura et al., 1995; De Ferrari et al., 1995). However, it must be noted, under certain circumstances (adrenergic stimulation mediated sudden [Ca^2+^]_i_ changes), Ca^2+^ release during an EAD is not governed by sarcolemmal Ca^2+^ influx, so that it is spontaneous, which resembles as a heterogenous pattern, just like in the case of DADs (Volders et al., 1997).

In our previous work, EADs were evoked by I_Kr_ blockade (dofetilide), activation of Na+ current (I_Na,L_) (veratridine), and activation of I_Ca,L_ (BAY K8644) at slow pacing rates. Additional application of the Ca^2+^ chelator BAPTA-AM decreased [Ca^2+^]_i_ as expected, but either reduced EAD frequency in the presence of dofetilide and veratridine or further increased EAD frequency in the presence of BAY K8644 (direct augmentation of the I_Ca,L_ brings extra Ca^2+^ inflow and is a substrate for increased EAD likelihood). Since BAPTA-AM decreased EAD frequency in the presence of veratridine, but failed to shorten APD, these results contradicts the exclusive role of APD in EAD generation and indicate that an increase in [Ca^2+^]_i_ is a significant factor not only for generating DADs, but for evoking EADs as well (Horvath et al., 2015). Moreover, in another set of experiments of Kistamas et al. H_2_O_2_ significantly increased APD and relative short term beat-to-beat variability (SV) (Kistamas et al., 2015a) and increased the occurrence of EADs on canine ventricular myocytes. Elevation of [Ca^2+^]_i_ in H_2_O_2_ was shown by others which can account for the increased SV and EAD incidence (Goldhaber, 1996; Xie et al., 2009; Szentandrassy et al., 2015; Kistamas et al., 2015). Furthermore, we also showed in guinea pig cardiomyocytes, that spontaneous Ca^2+^ release from the SR mediates (I_Na,L_) induced EADs (Horvath et al., 2013). The two possible mechanisms proposed by Zaza et al. by which I_Na,L_ promotes EAD genesis are (1) the reactivation of I_Ca,L_ during the plateau phase of AP and (2) SR Ca^2+^ overload (Zaza et al., 2008). In our experiments the first EAD occurred at a membrane potential more positive than the window Ca^2+^ current voltage range, meaning that not the reactivation of I_Ca,L_ was responsible for the generation of EADs. In fact, several mechanisms were addressed, showing the SR load was key in formation of the EADs: (a) anemone toxin II (ATX-II) facilitates I_Na,L_ that caused elevated systolic Ca^2+^ transient and SR load, (b) the spontaneous Ca^2+^ wave precedes the first EAD, and (c) Ca^2+^ buffering with BAPTA in the patch pipette abolished EADs (Horvath et al., 2013).

Therefore, our recent knowledge about the factors involved in the development of EADs includes changes in [Ca^2+^]_i_ and the amplitude of Ca^2+^ transient, along with the APD and beat-to-beat variability of APD, AP morphology and plateau potential, net membrane current, and the actual availability of L-type Ca^2+^ channels. Regardless of the type of EAD mechanisms, if the depolarizing effect of the EAD on the membrane potential is sufficient to activate I_Na_, the result will be an abnormal impulse generation, triggered activity (Hoffman and Rosen, 1981).

EADs are more likely to develop in midmyocardial cells and Purkinje fibers than in subepi- or subendocardial cells. There is a difference in ion current composition (less I_Ks_, more I_Na,L_ in midmyocardial cells), consequently these regions are more prone to AP prolongation (Liu and Antzelevitch, 1995; Zygmunt et al., 2001; Szabo et al., 2005). EADs are generally observed under conditions of ventricular hypertrophy and HF, injured cardiac tissue, or when the myocardium is exposed to catecholamines, hypoxia, acidosis, and pharmacologic agents (Roden, 2004; Roden, 2006). The clinical significance of EADs is clear, as they can either serve as the trigger or as the substrate for initiation and perpetuation of torsade de pointes arrhythmia (Volders et al., 2000). Being as a trigger, as EADs can cause new APs which will be reflected on the ECG as ectopic beats. EADs provide a substrate by causing electrical inhomogeneity in the surrounding tissues.

#### Delayed Afterdepolarization

DADs are the other common causes of focal activity and were originally described as oscillatory afterpotentials (Ferrier et al., 1973). They occur in diastole, after complete repolarization of the cell (**Figure 5B**). DADs can originate from intracellular Ca^2+^ overload that induces spontaneous SR Ca^2+^ release, resulting in a depolarizing current *via* forward mode I_NCX_ (Mechmann and Pott, 1986). Other nonselective Ca^2+^-sensitive cationic currents (I_NS_) and chloride current (I_Cl(Ca)_) may also be involved in DAD generation (Asakura et al., 2014). These three depolarizing currents result in a transient inward current (I_ti_), which is responsible for the membrane depolarization (**Figure 3D**). Ca^2+^ overload of the cardiac myocytes can occur in several diseases and also in several experimental conditions, e.g. toxic levels of digitalis (Ferrier et al., 1973; Saunders et al., 1973; Rosen et al., 1973), catecholamines (Wit and Cranefield, 1977; Rozanski and Lipsius, 1985; Priori and Corr, 1990), hypokalemia and hypercalcemia (Tse, 2016), hypertrophy, HF (Aronson, 1981; Vermeulen et al., 1994), and rapid heart rates. The amplitude of the generated DAD depends on the size of the Ca^2+^ transient and on the properties of I_NCX_ and the inward rectifier K^+^ current (I_K1_) (Pogwizd et al., 2001; Sung et al., 2006; Maruyama et al., 2010). Subthreshold DADs [appearing as the U wave on the electrocardiogram (ECG)] are small voltage deflections, which although unable to trigger a propagating action potential, may still cause dispersion of excitability, thereby promoting regional conduction block (Rosen et al., 1975; Surawicz, 1998; di Bernardo and Murray, 2002). However, if DADs reach the threshold potential for the opening of Na^+^ channels, a spontaneous AP emerges and can result in premature ventricular contraction (PVC). The clinical significance of DAD generation lies in triggered activity that contributes to arrhythmogenesis with catecholaminergic polymorphic ventricular tachycardia (CPVT), atrial fibrillation (AF), and HF. In CPVT and HF, intracellular Ca^2+^ load combines with RyR dysfunction (“leaky” RyR). Under circumstances when the SR becomes loaded (high Ca^2+^ load, fast heart rate, and/or increased adrenergic tone) and/or RyR becomes leaky, spontaneous Ca^2+^ release is favored.

Considering the mechanism of the spontaneous Ca^2+^ release, there are two main patterns. First, focal Ca^2+^ release, when Ca^2+^ signal acts locally (Lipp and Niggli, 1994) and secondly, when the released Ca^2+^ leaves its focus and propagates as a global Ca^2+^ wave through the myocyte (Takamatsu and Wier, 1990; Wier et al., 1987; Cheng et al., 1993).

Unlike the EADs, DADs are always generated at relatively rapid rates (Antzelevitch and Burashnikov, 2011). As mentioned earlier, late-phase 3 EADs are considered as a hybrid between EAD and DAD. A key difference is the time of the SR Ca^2+^ release during the AP (**Figure 5**). Ca^2+^ release occurs during diastole in the case of DAD, while late-phase 3 EAD is generated at the late repolarization of the AP (Fink and Noble, 2010).

#### Beat-To-Beat Variability of Action Potential Duration

Variations (physiological or pathological) in AP configuration can cause disturbances in Ca^2+^ signaling and the electrical properties of cardiac muscle. In our previous experiments, we determined the beat-to-beat variability of AP duration in isolated canine left ventricular myocytes in several experimental settings (Kistamas et al., 2015a; Kistamas et al., 2015b; Szentandrassy et al., 2015; Magyar et al., 2016), as recent studies suggest the short term beat-to-beat variability (SV) of APD as a novel method for predicting imminent cardiac arrhythmias (Thomsen et al., 2004; Abi-Gerges et al., 2010). Higher variability is considered to be arrhythmic by increasing dispersion of refractoriness (**Figure 3G**). We established the concept of relative short term beat-to-beat variability of APD (RSV) by normalizing the changes of short term variability of APD to the concomitant changes in APD [see (Nanasi et al., 2017] for review). We summarized that RSV was decreased by ion currents involved in the negative feedback regulation of APD (I_Ca,L_, I_Ks_ and I_Kr_), while it was increased by I_Na_ and I_to_, and in general, increased if repolarization reserved was compromised. RSV was also increased at faster rates and at increased [Ca^2+^]_i_. Transient changes of [Ca^2+^]_i_ due to Ca^2+^ released from the SR were the dominant contributor to this process (Kistamas et al., 2015b). High RSV at faster rates can also be explained by the elevated [Ca^2+^]_i_, as faster pacing increases I_Ca,L_, ultimately overloading the cell with Ca^2+^ which, in turn, increases RSV.

#### Cardiac Alternans

A severe form of this beat-to-beat variation is cardiac alternans, where short and long AP duration alternate (**Figure 3F**). Pulse and T-wave alternans can be clinically observed and are considered to be a precursor for cardiac arrhythmias (Rosenbaum et al., 1994; Verrier et al., 2011). Cardiac alternans originates from instabilities of membrane voltage or of Ca^2+^ cycling. At the cellular level, alternans is manifested as beat-to-beat alternations in contraction amplitude (mechanical alternans), APD (electrical or APD alternans), and Ca^2+^ transient amplitude (Ca^2+^ alternans) at constant heart rate. However, because of the bidirectional information flow between membrane voltage and Ca^2+^ cycling, electrical alternans is always influenced by Ca^2+^ alternans, and vice versa (Weiss et al., 2006).

Two mechanisms have been described for Ca^2+^-driven alternans. One depends on the relationship between SR Ca^2+^ content and the amount of Ca^2+^ released from the SR (Eisner et al., 2000). If this relationship is steep then a small increase of SR Ca^2+^ content will produce a large increase of the amplitude of the Ca^2+^ transient resulting in increased Ca^2+^ efflux *via* I_NCX_ and a decreased influx *via* I_Ca,L_ (Ca^2+^-dependent inactivation). The net result is a decrease of SR Ca^2+^ content. The next beat therefore arises from a depleted SR resulting in a smaller Ca^2+^ transient and decreased I_NCX_, so that the cell will gain Ca^2+^ resulting in a larger SR content and Ca^2+^ transient for the third beat (Eisner et al., 2006). Later, it was shown that reduced SERCA pump activity is also needed for an alternating pattern to develop (Shiferaw et al., 2003; Qu et al., 2007; Xie et al., 2008; Li et al., 2009). Another mechanism for Ca^2+^-driven alternans has been proposed, when on every beat, the SR load is unchanged, however the released amount of Ca^2+^ is alternating beat-to-beat. This kind of alternans results from the refractoriness of the RyRs, without the need for SR Ca^2+^ content alternans (Picht et al., 2006; Shkryl et al., 2012).

Voltage-driven or electrical alternans is determined by APD restitution. Here, the shorter the preceding diastolic interval, the less the APD (Nolasco and Dahlen, 1968). The steeper this relationship, the more likely is alternans to occur. There may be several causes for this APD restitution. The rapid, pacing-induced electrical alternans occurs at fast heart rates (short diastolic intervals, where recovery of I_Ca,L_ is crucial, becoming a key factor in regulating the steepness of APD restitution (Mahajan et al., 2008). Another APD alternating mechanism is driven by I_to_ at slow or normal heart rates and possibly accounts for T-wave alternans in patients with Brugada syndrome (Hopenfeld, 2006). The third type of electrical alternans is mediated by non-inactivating I_Ca,L_ with I_Ks_ at normal or slow rates and possibly cause T-wave alternans in LQTS patients (Wegener et al., 2008). Electrical, restitution-based alternans has been associated with the breakdown of reentry into ventricular fibrillation (VF). At the tissue level, if cellular alternanses in different regions of the ventricle occur in phase with each other (spatially concordant), T-wave alternanses is observed on the ECG. A more malignant form, the spatially discordant APD alternans, manifesting as QRS alternans on the ECG, causes large dispersion of refractoriness, a substrate for reentry. Spatially discordant alternans is a significant cause of wave break, a phenomenon that is essential to VF (Garfinkel, 2007). It has been shown, that interventions that lower the slope of the APD restitution curve can turn multiwave VF to single-wave monomorphic ventricular tachycardia (VT) (Garfinkel et al., 2000; Wu et al., 2002).

### Abnormal Impulse Conduction

Abnormal impulse conduction, i.e. reentry, occurs when the AP fails to terminate and has the ability to re-excite tissue regions which have already recovered. This mechanism can be divided into two main types, one with an obstacle (circus type with anatomical or functional barrier) and the other without an obstacle (phase-2 reentry and reflection). The key difference is in refractoriness. Circus movement reentry travels around an anatomic or functional obstacle and all cells are recovered from inactivation, while cells involved in reflection or phase-2 reentry show large differences in recovery from refractoriness with no obstacle in the way of the reentrant wave. In addition, classic nomenclature distinguishes between microreentry and macroreentry, where the reentrant circuit does not or does appear on the surface ECG, respectively.

**Figure 6:**
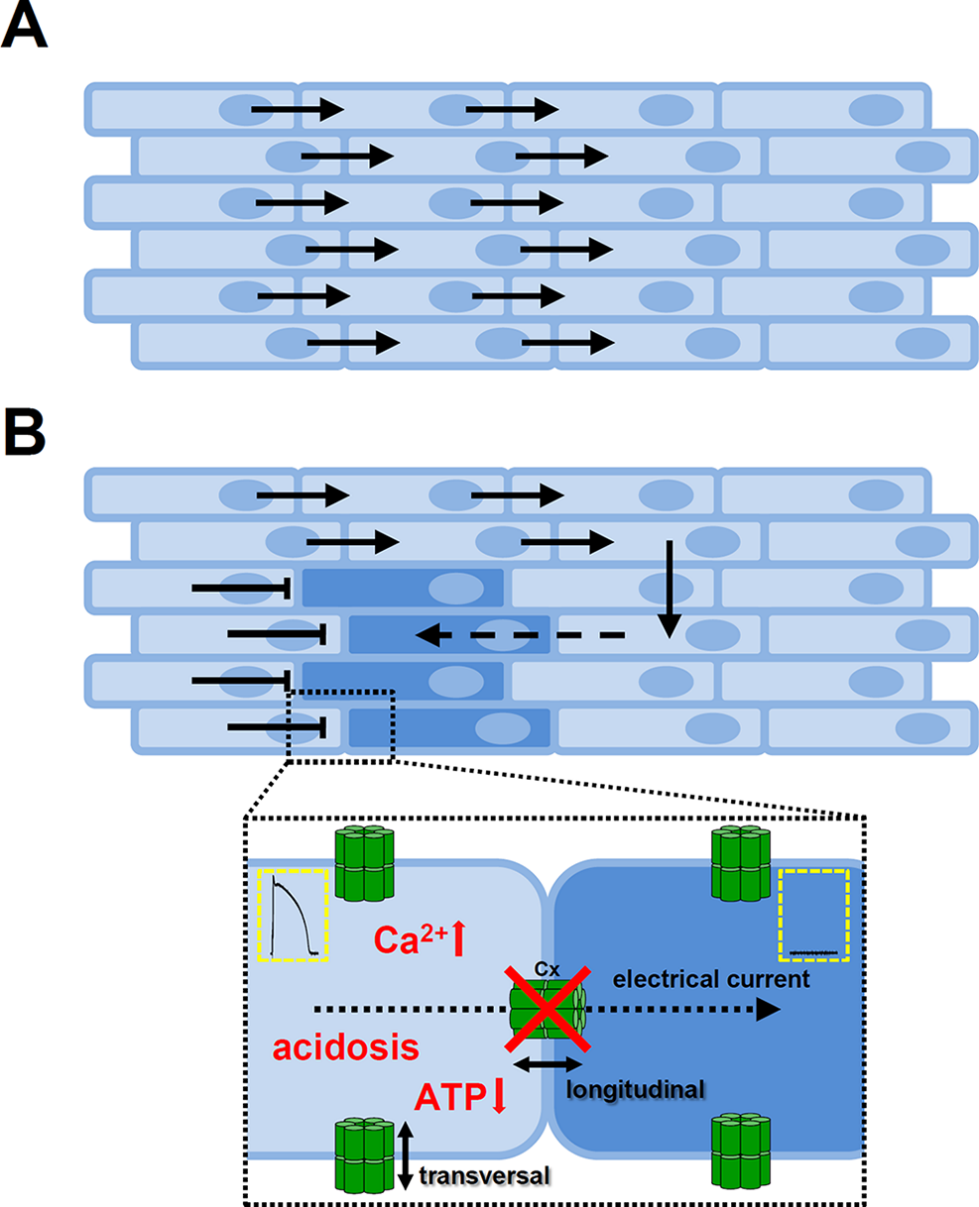
Role of gap junctions in propagating of the cardiac action potential. **(A)** The cardiac tissue is eletrically homogenous if the adjacent cells are coupled by gap junction channels. The anisotropic nature of gap junction channel distribution favors longitudinal over transversal conduction. **(B)** Conditions that decrease or abolish coupling between the cells may cause a unidirectional conduction block and as the electrical impulse propagates around the block it can re-excite those tissue regions due to differences in refractoriness. Insert shows cell-cell connections *via* gap junction channels. The main causes of uncoupling of the cells (showed in red) are elevated intracellular Ca^2+^ concentration, reduction in H^+^ concentration, or lower levels of ATP. Cx, connexon; ATP, adenosine triphosphate.

The myocardium works as a functional syncytium (**Figure 6A**). The elemental components of this system are the gap junctions. Gap junctions form channels (comprised of two neighboring connexons) between adjacent cardiomyocytes and allow the cardiac AP to propagate from cell to cell and thereby initiate contraction. However, gap junction channels are unevenly distributed within the cells, expressing a larger portion of channel proteins at the longitudinal ends of the cells than at the transversal, lateral sides (De Maziere and Scheuermann, 1990; Oosthoek et al., 1993). This anisotropy allows a much larger longitudinal conduction velocity and effective electrical coupling between the adjacent cells (**Figure 6B**). Several conditions are reported to reduce or abolish gap junctional conductance, including increased [Ca^2+^]_i_, reduced pH, or lower ATP levels (Dhein, 1998). Uncoupling of the cells may lead to the formation of unidirectional conduction block and reentry type arrhythmias (**Figure 6B**). The hypothesis that Ca^2+^ overload conditions have arrhythmogenic behavior is also supported by experiments in neonatal rat myocytes, where gap junctional conductance was decreased by Ca^2+^ concentrations higher than physiological (Firek and Weingart, 1995), while it was proposed that elevation of [Ca^2+^]_i_ by Ca^2+^ entry was more effective in decreasing gap junctional conductance than Ca^2+^ released from internal stores (Lazrak et al., 1994; Chanson et al., 1999). Furthermore, adequate coupling between the cells in the tissue (i.e. low longitudinal resistance) can suppress differences in APD, eliminate EADs, and reduce beat-to-beat variability (Magyar et al., 2015).

In the subsequent sections reentry types are discussed in detail.

#### Reentry With Anatomical Obstacle (Ring Model)

**Figure 7:**
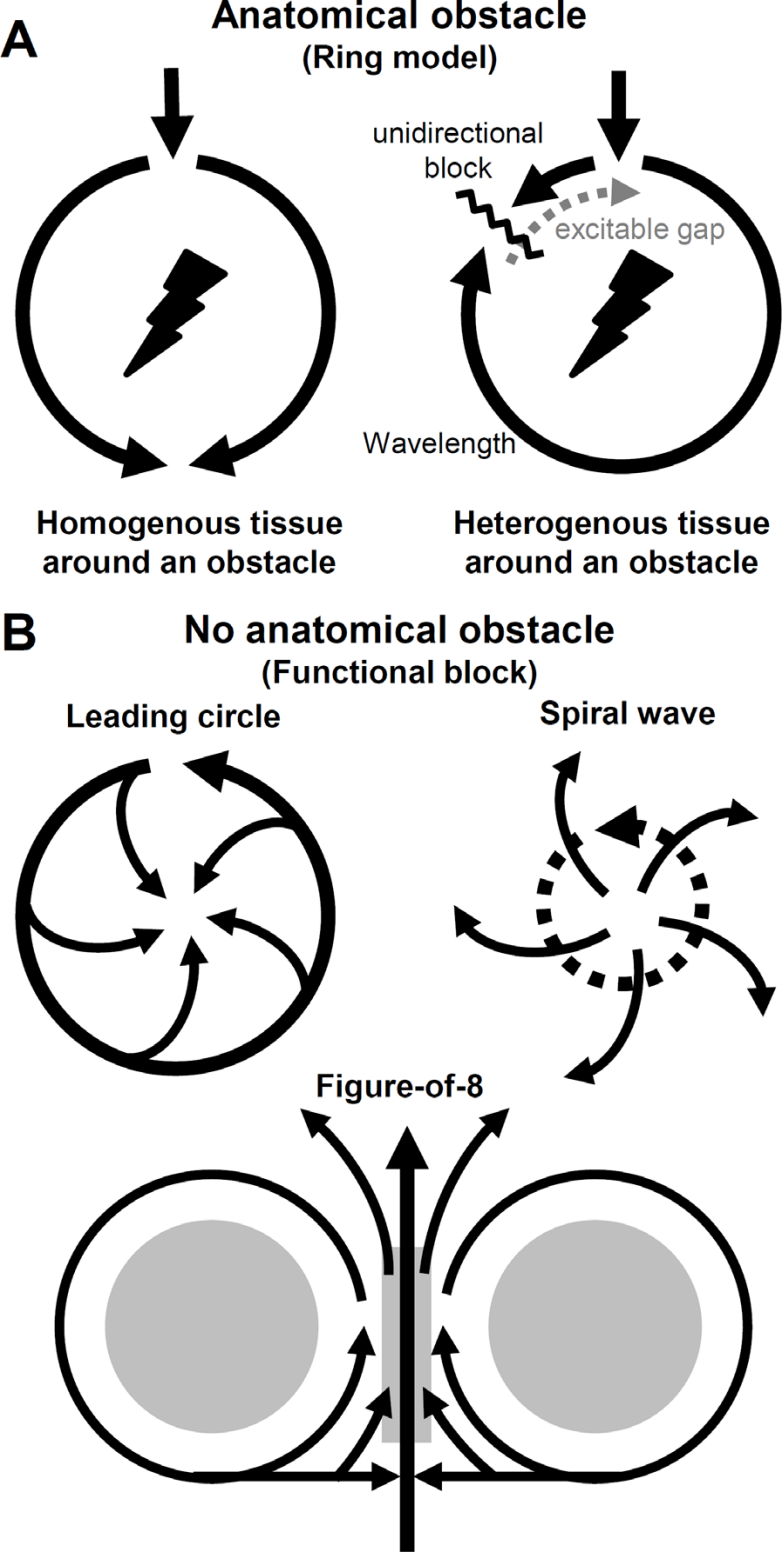
Abnormal impulse conduction. Circus movement reentry types. **(A)** Reentrant wave travels around an anatomical obstacle. If the cardiac tissue around the obstacle is homogenous the impulse conduction is favored in both directions. However, if the tissue is heterogenous (i.e. dispersion of refractoriness), unidirectional block can form initating a reentry circuit. Excitable gap consists of tissue regions that fully and/or partially recovered from refractory period, therefore excitable. **(B)** Circuit movement reentry can form in the absence of an anatomical obstacle (functional block). In the leading circle model the length of the circuit is not determined by the pathway around an obstacle, but rather by conduction velocity, refractory period, and stimulating efficacy where (in the absence of an obstacle) a shortcut of the circuit is possible. Spiral waves reentry (or scroll wave if three-dimensional) drifts through the tissue without an obstacle and the main wave can break up and radiate waves to the neighboring regions. In the model of figure-of-8 the circulating waves appear in pairs and the wavefront can circulate around the functional blocks clockwise and counterclockwise. If the intermediate area (central gray) can be activated by the colliding separated waves, reentry can form.

Reentry was first described in 1906 by Mayer in rings of tissue cut from jellyfish (ring model) (Mayer, 1906). Later work by Mines showed that circus-type reentry can be initiated by electrical stimulation in cardiac muscle and was the first to define the concept of circus movement reentry around an anatomical obstacle (**Figure 7A**) (Mines, 1913; Mines, 1914). The anatomical barrier can be a valve, vessel or scar. The possibility that circus-type reentry can form without an anatomical obstacle was proposed by Garrey (1914).

Initiation of reentry requires a trigger and a substrate. The trigger can be a premature contraction, while tissue substrate is the dispersion of refractoriness. On top of that, fundamental settings are needed for reentry excitation with anatomical obstacle: (1) the impulse initiating the circus movement must propagate in one direction (unidirectional block) and (2) the proportion of absolute and relative refractoriness in the tissue, that is, the reentrant circuit must be long enough to let all areas—within the circuit, distal from the stimulus—recover from refractory (excitable gap), so the circuit can return to its origin and continue as a new cycle (**Figure 7A**). Consequently, (3) the circulating movement would terminate in case of interruption of the reentrant circuit (Mines, 1913). These criteria proposed by Mines are still in use today. The above mentioned excitation is, in fact, a propagating wave. The length of this wave (wavelength) is determined by the distance between the wavefront (phase 0, AP depolarization) and waveback (phase 3, repolarization), that is, creating an arrhythmogenic excitation needs the special properties of refractoriness and conduction velocity (Weiss et al., 2000). If the above three criteria are not met, i.e. in sinus rhythm if the tissue around the anatomical obstacle is homogenous (and the impulse pathway is wide enough), the wavefront can simultaneously propagate in both pathways around the barrier. However, if the tissue is electrically heterogenous, due to dispersion of refractoriness, unidirectional conduction block can form caused by a PVC, i.e. initiating reentry (**Figure 7A**).

#### Reentry Without Anatomical Obstacle (Functional Block)

In the cases, when there is no anatomical barrier present, functional reentry can still form, maintained only by the electrical properties (dispersion of refractoriness) of the tissue. The best known examples are the leading circle, spiral wave, and figure-of-8 reentry (**Figure 7B**).

The leading circle model was described by Allessie et al., as “the head of the circulating wavefront is continuously biting in its own tail of refractoriness” (Allessie et al., 1977). The main differences compared to the ring model are (1) the length of the circuit is determined by conduction velocity, stimulating efficacy, and refractory period not by an anatomic obstacle, (2) while the length of the circuit is not fixed, it can be altered by changes in electrophysiological properties of the tissue. (3) There is no excitable gap in the leading circle model and (4) a shortcut of the circuit is possible and finally (5) revolution time is proportional to refractory period, while in the ring model, revolution time is inversely related to conduction velocity (**Figure 7B**) (Allessie et al., 1977).

Spiral waves and rotors can be induced in small two-dimensional pieces of cardiac muscle, without an anatomical barrier, and can drift through the tissue (Pertsov et al., 1993). Scroll waves are the three-dimensional forms of spiral waves. Spiral waves can develop both in homogenous and heterogenous tissues and either in stable or in an unstable form (Ikeda et al., 1996; Davidenko et al., 1992). The former might result in monomorphic VT, while the latter can cause polymorphic VT or torsade de pointes (**Figure 7B**) (Gray et al., 1995).

Figure-of-8 type reentry was first demonstrated by el-Sherif et al. In this case the reentrant wavefront reaches a functional conduction block surrounded by regions of reduced excitability. As conduction is not favored through such tissue, the wavefront drives clockwise and counterclockwise around the two arcs of functional block and beyond the barriers of low excitability the two separated waves can collide. If the conduction is slow enough and the intermediate area can be activated, reentry can form (**Figure 7B**) (el-Sherif et al., 1985; Lazzara, 1988).

#### Phase-2 Reentry

In the previous reentrant mechanisms, the trigger and the substrate originated from different etiologies, while in the case of phase-2 reentry, trigger and substrate are from the same source. Phase-2 reentry occurs in ischemia (Lukas and Antzelevitch, 1996), Brugada syndrome (Brugada and Brugada, 1992) or under conditions of higher pacing rates and higher extracellular Ca^2+^ concentration (Di Diego and Antzelevitch, 1994). It is caused by severe spatial dispersion of repolarization, that is, spike-and-dome configuration of AP morphology is lost at one site (predominantly at the epicardial region), while preserved at another site and is responsible for the transition to VT and VF. APs without the dome (short APD, early repolarization) can therefore be reexcited and reentry can be initiated (Antzelevitch, 2007). Loss of dome can be explained by a stronger transient outward current (I_to_) current, and overall by the competitive behavior between I_Na_ and I_to_ (Greenstein et al., 2000; Szabo et al., 2005; Dong et al., 2010). If the actual membrane potential value is more negative than the activation threshold for the I_Ca,L_ then the AP dome vanishes. Cantalapiedra et al. showed in a simplified ionic and in a realistic cardiac model, that the origin of reexcitation is based on the presence of slow Ca^2+^ pulse, produced by the slow inward Ca^2+^ current (I_si_), so that the slow pulse propagates to the regions of short APs until it triggers a fast pulse (Cantalapiedra et al., 2010). Interestingly, the same research group argued that conditions (e.g. drugs) increasing the I_Ca,L_, to recover the dome or to prevent the loss of dome, decreases dispersion of repolarization, however, also increasing the probability of reexcitation, through the stabilizing effect of the Ca^2+^ conductance (I_Ca,L_) on the slow Ca^2+^ pulse (Cantalapiedra et al., 2009).

#### Reflection

Reflection is another example of non-circus movement reentry, with a one-dimensional behavior and can be the cause of PVCs or even lethal arrhythmias (Wit et al., 1972; Rosenthal, 1988; Van Hemel et al., 1988). Reflection describes reentry in a linear bundle of a conductive tissue. A stimulus from the proximal region travels through an inexcitable gap and elicits an AP at the distal end. Slow electrotonic currents (inexcitable region can only transmit electrotonic currents) generated by this AP can then propagate in the retrograde direction and reenter and reexcite the proximal elements (Antzelevitch et al., 1980). There must be an adequate conduction delay to let reflection happen (proximal end can recover from refractoriness), depending on the pacing interval and stimulus strength. It was also shown that neither EADs nor automaticity was required for reflection (Cabo and Barr, 1992; Kandel and Roth, 2015).

#### Biexcitability

A novel wave dynamic, termed biexcitability has been described in recent studies (Chang et al., 2012). In pacemaker regions I_Ca,L_ causes the activation, while in working muscle cells, the upstroke of the AP is driven by I_Na_ and I_Ca,L_. During biexcitability both form of activation can coexist at the same tissue. Under certain conditions, like long QT syndrome, repolarization reserve is compromised, APD prolongs, and EADs can occur. Consequently, there can be a situation where the cells develop two stable membrane potential values (−80 mV and −50 mV) and switches between them (Gadsby and Cranefield, 1977), resulting in a Na^+^- and Ca^2+^-mediated (fast) or a Ca^2+^-mediated (slow) propagating wavefront. This bi-stable behavior might serve as an explanation for the two different possible outcomes of torsade de pointes. According to Chang et al., in cases where the Ca^2+^-mediated slow spiral wave is terminated, leads to termination of the torsade de pointes, while if the tissue is sufficiently heterogenous, Na^+^ and Ca^2+^-mediated fast spiral waves degenerate torsade de pointes to VF (Chang et al., 2012; Chang et al., 2013).

DADs can induce focal VT by DAD-mediated triggered activity or initiate reentry. Moreover, unstable Ca^2+^ signaling can dynamically serve as a substrate for reentry, by promoting dispersion of excitability or promoting dispersion of refractoriness (Weiss et al., 2015). In those tissue regions, where subthreshold DADs do not trigger a propagating AP, the resultant small membrane depolarization can still be sufficient to depress excitability by inactivating the fast voltage gated Na^+^ channels. This condition can lead to reentry, as the inactivated Na^+^ channels form a regional conduction block for impulses generated by suprathreshold DADs (Rosen et al., 1975; Liu et al., 2015). In the latter case, DAD-mediated triggered activity at fast rates can promote Ca^2+^ transient alternans, which in turn causes APD alternans, thereby increasing the dispersion of refractoriness (Sato et al., 2006; Weiss et al., 2006). As previously mentioned, subthreshold EADs can also enhance the dispersion of refractoriness, also creating a reentry substrate.

For more detailed reviews on conduction disorders, see Qu and Weiss (2015) and Antzelevitch and Burashnikov (2011).

The following sections will provide further insights into intracellular Ca^2+^ handling maladies in the most prevalent inherited and acquired arrhythmia syndromes, caused by channelopathies and defects in Ca^2+^ handling genes. Ca^2+^ handling defects also have an arrhythmogenic role in diseases, such as heart failure and cardiomyopathies, however they are beyond the scope of the present review [see recent reviews (Coppini et al., 2018; Johnson and Antoons, 2018; Denham et al., 2018)].

## Inherited Syndromes

### Catecholaminergic Polymorphic Ventricular Tachycardia

Catecholaminergic Polymorphic Ventricular Tachycardia (CPVT) is a severe arrhythmogenic disorder, manifesting as a bidirectional or polymorphic VT, mainly in young patients with structurally healthy hearts after exercise or acute emotional stress (Reid et al., 1975). As heart rate increases as a result of exercise or emotional stress, the ectopic ventricular trigger increases in complexity, such that VT turns into VF and may lead to syncope or sudden cardiac death (Coumel, 1978; Leenhardt et al., 1995).

The main criteria for CPVT diagnosis are as follows: structurally normal heart (and normal coronary arteries in individuals above 40 years of age), normal QT interval, and adrenergic induced bidirectional or polymorphic VT (Venetucci et al., 2012). CPVT is also diagnosed in patients who carry a pathogenic mutation and in family members of a CPVT index case, fulfilling the above mentioned criteria (Priori et al., 2013). There are also nonspecific features, therefore not diagnostic criteria, including a prominent U wave on the ECG accompanied by sinus bradycardia (Postma et al., 2005).

In CPVT, arrhythmias are induced by Ca^2+^ release from the SR leading to a DAD. The fundamental feature of this process is the Ca^2+^ release unit (Ca^2+^ sparks), where the spontaneous Ca^2+^ release occurs. If sufficient number of release units are activated, a Ca^2+^ wave is born, which depends on the SR Ca^2+^ content and the SR Ca^2+^ threshold (Lukyanenko et al., 1999; Venetucci et al., 2007). Interventions that alter RyR opening will affect SR Ca^2+^ threshold. For example, caffeine increases the open probability of RyR, therefore it is easier to elicit spontaneous Ca^2+^ release (Trafford et al., 2000), on the other hand tetracaine has an opposite effect, by reducing RyR opening, SR Ca^2+^ release threshold is higher (Overend et al., 1997; Venetucci et al., 2006).

In the previous sections we detailed the normal Ca^2+^ cycling and consequences of elevated [Ca^2+^]_i_. Briefly, the main arrhythmogenic mechanism in CPVT is due to SR Ca^2+^ release increasing cytoplasmic Ca^2+^ levels, NCX exchanges Ca^2+^ with Na^+^, thereby generating I_ti_. I_ti_ produces DADs and if DADs reach the activation threshold of Na^+^ channels, an elicited AP causes triggered activity, which in turn can lead to an extrasystolic heartbeat. Mutations in CPVT have been shown to alter RyR function and increase the occurrence of spontaneous Ca^2+^ release events after sympathetic stimulation (Liu et al., 2006). β-adrenergic activation increases SR Ca^2+^ content, while the same process enhances RyR phosphorylation by Ca^2+^/calmodulin-dependent protein kinase II (CaMKII) and protein kinase A (PKA) (Kashimura et al., 2010; Liu et al., 2011a; Venetucci et al., 2012). In addition to the phosphorylation by PKA, CaMKII-mediated phosphorylation increases the I_Ca,L_ and SERCA (by removing the inhibitory effect of phospholamban on SERCA) and activates RyR. Simultaneous activation of I_Ca,L_, SERCA (increases SR Ca^2+^ content), and RyR therefore increases the possibility of spontaneous Ca^2+^ release (Maier and Bers, 2007; Hegyi et al., 2019). Experimental data confirmed that higher RyR Ca^2+^ sensitivity alone is not sufficient to elicit spontaneous Ca^2+^ release and that inhibition of CaMKII in a CPVT mouse model prevents arrhythmias (Venetucci et al., 2007; Liu et al., 2011a).

Several CPVT subtypes have been described to date, albeit the two most common types are the CPVT-1 and CPVT-2 (**Table 1**).

**Table 1:** Ca^2+^ handling genes associated with inherited arrhythmogenic syndromes.

Syndrome/ Phenotype	Genes	Genetic Locus	Functional effect	Protein	Ref	Syndrome overlap
**CPVT-1**	RYR2	1q43	GoF	**ryanodine receptor 2**	(Laitinen et al., 2001; Priori et al., 2001)	
**CPVT-2**	CASQ2	1p13.1	GoF	**calsequestrin 2**	(Lahat et al., 2001)	
**CPVT-4**	CALM1	14q31–q32	LoF	**calmodulin 1**	(Nyegaard et al., 2012; Sondergaard et al., 2015; Sondergaard et al., 2017)	LQTS-14
**CPVT-5**	TRDN	6q22.31	LoF	**triadin**	(Chopra et al., 2009)	LQTS-17
**LQTS-4**	ANK2	4q25–q26	LoF	**ankyrin B**	(Bhuiyan et al., 2013; Mohler et al., 2003)	
**LQTS-8** **(Timothy syndrome)**	CACNA1C	12p13.33	GoF	**α1_C_ subunit of LTCC**	(Splawski et al., 2004; Thiel et al., 2008; Boczek et al., 2015; Landstrom et al., 2016)	BrS-3, SQTS-4, ERS/IVF
**LQTS-14**	CALM1	14q32.11	GoF/LoF	**calmodulin 1**	(Shamgar et al., 2006; Gray and Behr, 2016; Jensen et al., 2018; Wren et al., 2019)	CPVT-4
**LQTS-15**	CALM2	2p21	LoF	**calmodulin 2**	(Shamgar et al., 2006; Gray and Behr, 2016; Jensen et al., 2018; Wren et al., 2019)	
**LQTS-16**	CALM3	19q13.32	LoF	**calmodulin 3**	(Shamgar et al., 2006; Gray and Behr, 2016; Jensen et al., 2018; Wren et al., 2019)	
**LQTS-17** **(Triadin Knockout Syndrome)**	TRDN	6q22.31	LoF	**triadin**	(Altmann et al., 2015)	CPVT-5
**BrS-3**	CACNA1C	12p13.33	LoF	**α1_C_ subunit of LTCC**	(Schwartz et al., 1995; Rosero et al., 1997)	SQTS-4, ERS/IVF
**BrS-4**	CACNB2	10p12.33–p12.31	LoF	**β_2_ subunit of LTCC**	(Schwartz et al., 1995; Rosero et al., 1997)	SQTS-5, ERS/IVF
**BrS-11**	CACNA2D1	7q21.11	LoF	**α_2_δ_1_ subunit of LTCC**	(Schwartz et al., 1995; Rosero et al., 1997)	SQTS-6, ERS/IVF
**BrS-15**	TRMP4	19q13.33	GoF/LoF	**transient receptor potential melastatin 4**	(Liu et al., 2013)	
**SQTS-4**	CACNA1C	12p13.33	LoF	**α1_C_ subunit of LTCC**	(Antzelevitch et al., 2007; Bjerregaard et al., 2010)	BrS-3, ERS/IVF
**SQTS-5**	CACNB2	10p12.33–p12.31	LoF	**β_2_ subunit of LTCC**	(Antzelevitch et al., 2007; Bjerregaard et al., 2010)	BrS-4, ERS/IVF
**SQTS-6**	CACNA2D1	7q21.11	LoF	**α_2_δ_1_ subunit of LTCC**	(Antzelevitch et al., 2007; Bjerregaard et al., 2010)	BrS-11, ERS/IVF
**ERS/IVF**	CACNA1C	12p13.33	LoF	**α1_C_ subunit of LTCC**	(Priori et al., 2013)	BrS-3, SQTS-4
	CACNB2	10p12.33–p12.31	LoF	**β_2_ subunit of LTCC**	(Priori et al., 2013)	BrS-4, SQTS-5
	CACNA2D1	7q21.11	LoF	**α_2_δ_1_ subunit of LTCC**	(Priori et al., 2013)	BrS-11, SQTS-6

CPVT, catecholaminergic polymorphic ventricular tachycardia; LQTS, long QT syndrome; BrS, Brugada syndrome; SQTS, short QT syndrome; ERS, early repolarization syndrome; IVF, idiopathic ventricular fibrillation; LTCC, L-type Ca^2+^ channel; GoF, gain-of-function; LoF, loss-of-function.

CPVT-1 is caused by an autosomal dominant mutation in the RyR2 gene (Swan et al., 1999). This subtype is the most common, accountable for about 60% of all CPVT cases (Laitinen et al., 2001; Priori et al., 2001). RyR exists as a macromolecular complex with many other molecules, such as calsequestrin 2 (CSQ2), FK506 binding protein 1B (FKBP1B or FKBP12.6), FK506 binding protein 1B (FKBP1B or FKBP12.6), PKA, CaMKII, phosphatase 1 (PP1), phosphatase 1 (PP1), phosphatase 2A (PP2A), histidine-rich Ca^2+^ binding protein (HRC), junctin and triadin (Wang et al., 1998; George et al., 2007; Yano et al., 2009; Arvanitis et al., 2011; Szabo et al., 2013). Junctin and triadin mediates interaction between RyR and CSQ2 (Eisner et al., 2017). Most RyR mutations in CPVT are gain-of-function mutations and thereby leading to increased Ca^2+^ sensitivity and RyR channels may open during diastole causing Ca^2+^ leak, particularly during adrenergic stress (Jones et al., 2008). Several hypotheses have been advanced to explain this phenomenon, including the role of FKBP12.6, store overload-induced Ca^2+^ entry (SOCE) and a defective mutation in the RyR 3D conformation (Reiken et al., 2003; Lehnart et al., 2004; Jiang et al., 2005; Yamamoto et al., 2008; Liu et al., 2009; Uchinoumi et al., 2010; Suetomi et al., 2011; Venetucci et al., 2012a).

CPVT-2 is an autosomal recessive gene anomaly in CASQ2-encoded CSQ2 and responsible for about 3–5% of CPVT patients (Lahat et al., 2001). The structure of this intra-SR Ca^2+^ buffer changes Ca^2+^ concentration. At low SR Ca^2+^ concentrations (< 0.6 mmol/L) CSQ2 is a monomer, which is converted to a dimer (0.6–3 mmol/L) or polymer (> 3 mmol/L) at higher Ca^2+^ concentrations (Mitchell et al., 1988; Wang et al., 1998). It has been shown that, in the absence of functional CSQ2, RyR channels open spontaneously, without the need for L-type Ca^2+^ current mediated trigger (Knollmann et al., 2006) and that mutation of CSQ2 destabilizes Ca^2+^ storing capacity of the SR, which in turn alters the Ca^2+^ sensitivity of RyR (Viatchenko-Karpinski et al., 2004). In all CSQ2 mutations (missense, deleterious, nonsense), level of CSQ2 protein is reduced or absent, perhaps because it is more susceptible to degradation (Rizzi et al., 2008; Faggioni et al., 2012). Impaired polymerization (Bal et al., 2010), reduced RyR binding and modulation (Houle et al., 2004; Terentyev et al., 2006) are generally associated with lower SR Ca^2+^ content, higher [Ca^2+^]_i_ and Ca^2+^ leak through RyR, these effects can be augmented by β-stimulation (Song et al., 2007). An interesting feature of CSQ2 protein reduction is a subsequent reduction in triadin and junctin levels. Denegri et al. showed in CSQ2 knock-out animal model that viral gene transfer for *in vivo* replacement of CSQ2 restored normal CSQ2 levels along with triadin and junctin, and ultimately prevented arrhythmias (Denegri et al., 2012).

Other, less frequent gene mutations have also been described, such as autosomal recessive forms of CPVT, the CPVT-3 and CPVT-5, while CPVT-4 is an autosomal dominant form of the inherited syndrome. CPVT-3 subtype is related to the gene encoding trans-2,3-enoyl-CoA reductase-like protein (TECRL) and is first seen at an early age with high likelihood of infant sudden cardiac death (Bhuiyan et al., 2007). When CPVT-3 is studied in induced pluripotent stem cell-derived cardiomyocytes (iPSC-CM) slower Ca^2+^ reuptake, slower Ca^2+^ transient upstroke velocity, and increased APD has been observed, along with norepinephrine-induced DADs, which could be eliminated by flecainide (see below) (Devalla et al., 2016). Mutations in CALM1-encoded calmodulin (CaM) cause the CPVT-4 subtype. *In vitro* experiments showed that this gene anomaly in the C domain compromises Ca^2+^ binding to CaM and impairs interaction between RyR and its CaM-binding domain, leading to an increased open state of RyR (Nyegaard et al., 2012; Sondergaard et al., 2015; Sondergaard et al., 2017). TRDN-encoded triadin mutation results in CPVT-5 subtype, which may cause diastolic Ca^2+^ leak and Ca^2+^ overload. Electron microscopy experiments uncovered fragmentation and reduced contact at the dyadic cleft, thus possibly lacking the negative feedback of SR Ca^2+^ release on the L-type Ca^2+^ channels, so SR Ca^2+^ overload may arise from the uncontrolled Ca^2+^ influx (Chopra et al., 2009).

A possible loss-of-function RyR mutation has also been proposed in a case classified as idiopathic VF, where a reduced SR Ca^2+^ sensitivity was shown (Jiang et al., 2007). Moreover, exercise induced bidirectional VT has been reported in types of long QT syndromes (LQTS-4 and LQTS-7) (**Table 1**) (Mohler et al., 2004; Vega et al., 2009).

Because of the hiding nature of the disease, it is difficult to diagnose CPVT, as patients have normal heart structure and show no symptoms before syncope or sudden cardiac death. However, if diagnosed, there are several therapeutic approaches to CPVT.

Generally speaking, life-long administration of β-blockers is the first choice as treatment. Studies showed that nadolol was clinically effective and a useful prophylactic (Priori et al., 2013). In countries, where nadolol is not available, propranolol was also shown to be effective (Hayashi et al., 2009). Carvedilol has been shown to inhibit store overload-induced Ca^2+^ release (SOICR) and is the only β-blocker to have RyR inhibitory action, albeit it is a less potent β-blocker after all (Zhou et al., 2011). Patients with CPVT are recommended to remove the triggers, in other words to limit or avoid any vigorous physical activities and stressful environments (Priori et al., 2013). In some patients (lacking long-term studies yet) β-blocker and non–dihydropyridine Ca^2+^-channel blocker (verapamil) combination therapy was shown to be beneficial (Swan et al., 2005; Rosso et al., 2007).

Flecainide administration has been suggested on top of β-blockers to prevent arrhythmias, in CPVT patients refractory to β-blockers alone (Biernacka and Hoffman, 2011; Pott et al., 2011; van der Werf et al., 2011). Flecainide is a Na^+^-channel blocker drug, specifically a Class Ic antiarrhythmic agent. Several studies, including three retrospective cohorts in human patients with CPVT (Liu et al., 2011; Radwanski et al., 2016; Kannankeril et al., 2017) have shown the effectiveness of flecainide but there is still debate around the mechanism by which it exerts its antiarrhythmic effect. Watanabe et al. concluded that the most important effect of flecainide was blocking the RyR along with the Na^+^-channel blockade (Watanabe et al., 2009). They hypothesized that blocking RyR reduces the spontaneous Ca^2+^ release events and therefore DADs, while Na^+^-channel blockade prevents the possibility of triggered activity from any residual DADs (Hilliard et al., 2010). Of the Class Ic antiarrhythmic drugs, only flecainide and propafenone was shown to inhibit RyR activity (Hwang et al., 2011). On the other hand, Liu et al. showed in an animal model of CPVT that although flecainide prevents VT and triggered activity, spontaneous Ca^2+^ release and DADs were still detectable in single myocytes. They concluded that the antiarrhythmic effect of flecainide results from its Na^+^-channel blocker effect rather than *via* RyR inhibition (Liu et al., 2011b; Bannister et al., 2015). These conflicting results raise the question whether the different effects seen in the previous studies are dependent of a specific genetic mutation. In a recent study, isolated myocytes from Casq2^-/-^ and RyR2R4496C^+/-^ mice were compared (Hwang et al., 2019). It was found that the former produces a stronger proarrhythmic response upon isoproterenol stimulation, but flecainide prevented arrhythmias in both cases. Also independent from the underlying mutation, effect of flecainide decreased at high Ca^2+^ load. An additional drug has also been tested both *in vitro* and *in vivo*. 1,4-benzothiazepine derivative K201 (JTV519) was shown to prevent arrhythmias in mouse models by reducing RyR opening, SERCA activity and I_Ca,L_ (Lehnart et al., 2004; Loughrey et al., 2007).

The latest guidelines recommend implantable cardiac defibrillator (ICD) implantation in patients with diagnosis of CPVT who experience VT, syncope, or cardiac arrest despite the optimal medical treatment (Priori et al., 2013). However, the use of ICDs without concomitant use of β-blockers is dangerous because of the possibility of shock-related electrical storms in these patients (Mohamed et al., 2006; Pflaumer and Davis, 2012). Selective left cardiac sympathetic denervation (LCSD) can be a useful therapeutic method and may be considered in patients with uncontrollable arrhythmias (patients with contraindication to β-blockers; when ICD cannot be implanted; or when recurrent VTs manifest in patients with ICD and β-blockers treatment) (Priori et al., 2013). Pulmonary vein isolation (catheter ablation) was reported to be efficient in some patients with CPVT and AF (Sumitomo et al., 2010), while the possibility of gene therapy was suggested after successful adenoviral vector infection (CASQ2 gene) in R33Q knock-in mutant mouse with dysfunctional CSQ2 (Denegri et al., 2014). Family screening of first degree relatives (clinical evaluation and genetic testing) has been strongly suggested with an optional β-blocker therapy even in the absence of a positive exercise test (Bauce et al., 2002; Hayashi et al., 2009).

### Congenital Long QT Syndrome

Congenital long QT syndrome (LQTS) is an inherited cardiac ion channelopathy. LQTS is characterized by a prolonged QT interval on the surface ECG, reflecting the ventricular APD prolongation, which gives rise to risk for syncope, seizures, VT or torsade de pointes and finally VF and sudden cardiac death (Schwartz et al., 2012). Prolongation of APD can happen in an inhomogenous pattern, resulting in an enhanced dispersion of repolarization across the tissue. Delay in repolarization can occur e.g. by genetic defects of key ion currents, namely I_Ks_, I_Kr_, or I_Na_. As mentioned in a previous section, EADs can form if the repolarization reserve is compromised, outward currents are reduced and/or inward currents are increased. In the case of LQTS, inhomogeneity of refractoriness combined with EADs establishes the arrhythmia substrate for VT, torsade de pointes.

The above mentioned conditions are illustrated in the cases of LQTS-1, LQTS-2, and LQTS-3. LQTS-1 is caused by the loss-of-function mutation of KCNQ1 gene (K_v_7.1) that encodes I_Ks_ (Sanguinetti et al., 1996; Barhanin et al., 1996) while LQTS-2 is also a loss-of-function mutation, but of the KCNH2 channel gene (K_v_11.1), encoding I_Kr_ (Sanguinetti et al., 1995). LQTS-3 is an inherited gain-of-function mutation of SCN5A Na^+^ channel (Na_v_1.5) encoding I_Na_ (Wang et al., 1995). All three mutations play key role in determining the length of AP and all of them points towards compromised repolarization reserve with decreased outward currents (LQTS1-2) and increased inward current (LQTS-3). LQTS-1–3 account for ~75–85% of the congenital LQTS cases (El-Sherif et al., 2017).

Mutations of several other genes have been described in LQTS patients. Mutations of structural and channel interacting proteins result in: LQTS-4, a loss-of-function mutation of ANK2-encoded ankyrin B and leads to Ca^2+^ overload, QT prolongation, sinus bradycardia, AF, and CPVT (Bhuiyan et al., 2013; Mohler et al., 2003); LQTS-5, a loss-of-function KCNE1-encoded minK mutation, consequential reduction in I_Ks_ (Splawski et al., 1997); LQTS-6, a loss-of-function mutation of KCNE2-encoded MiRP1, causing a faster inactivation time course for I_Kr_, enhanced I_Ca,L_, and reduced I_f_ (Lu et al., 2003; Nawathe et al., 2013; Liu et al., 2014); LQTS-9, CAV3-encoded Caveolin 3, causing an enhanced I_Na,L_; and LQTS-11, a mutant A-kinase anchoring protein (AKAP9-Yotiao) results in an abnormal response upon β-stimulation, as mutation reduces interaction between AKAP9 and K_v_LQT1 cannel α subunit (KCNQ1, I_Ks_) leading to dysfunctional response to cAMP and a prolonged APD (QT) (Chen et al., 2007).

LQTS-9 and LQTS-10 (gain-of-function mutation in SCN4B-encoded Na^+^ channel Na_v_β4 β-subunit) together resemble the LQTS-3 phenotype as QT prolongation is achieved by increased Na^+^ current (Medeiros-Domingo et al., 2007). Mutation of SNTA1-encoded α1-syntrophin is a gain-of-function gene anomaly, causing LQTS-12 by enhancing Na^+^ current (Na_v_1.5) (Wu et al., 2008). LQTS-7 and LQTS-13 are affecting repolarizing K^+^ currents and channels. LQTS-7 or Andersen-Tawil type 1 syndrome is caused by the loss-of-function mutation of the KCNJ2-encoded K_ir_2.1 inward rectifier K^+^ channel, responsible for I_K1_, and as I_K1_ is an important player in terminal repolarization, reduction of K_ir_2.1 function prolongs QT interval (Plaster et al., 2001). In LQTS-13, a loss-of-function mutation on KCNJ5-encoded K_ir_3.4 causes loss of acetylcholine activated, G-protein-gated K^+^ (I_KAch_) channel function. I_KAch_ is formed by K_ir_3.1 and K_ir_3.4. Mutation in K_ir_3.4 function disrupts membrane targeting and stability, i.e. reduced membrane expression has been suggested as the cause of LQTS-13 (Yang et al., 2010).

Although most of the LQTS mutant genes are related to K^+^ and Na^+^ channels (i.e. LQTS-1–3 being ~75–85% of total congenital LQTS), there are several Ca^2+^-signaling proteins that are linked to the occurrence of long QT intervals, typically causing LQTS-8, LQTS-14, LQTS-15, LQTS-16, and LQTS-17 (**Table 1**).

LQTS-8 is a gain-of-function mutation of the CACNA1C-encoded α1_C_ subunit of L-type Ca^2+^ channel (Ca_v_1.2) and is generally associated with Timothy syndrome. Timothy syndrome is a rare (less than 30 patients reported worldwide), but severe multisystem disorder, involving QT prolongation, syndactyly, congenital heart defects, cardiomyopathies, bradycardia (caused by AV block rather than sinus bradycardia), and autism (Splawski et al., 2004). LQTS-8 mutation of the Ca_v_1.2 leads to (1) a significant reduction in voltage-dependent inactivation of I_Ca,L_, (2) enhanced I_Ca,L_, (3) decreased current density with enhanced window current, and (4) a steeper APD restitution curve (Thiel et al., 2008; Boczek et al., 2015; Landstrom et al., 2016). A lesser inactivation of the steady-state current and/or increased peak current means a higher Ca^2+^ influx, which can in turn prolong APD, therefore QT interval. A steeper APD restitution curve is proarrhythmic, being a substrate for alternans, as detailed in previous chapters. The mutation can also cause T-wave alternans on the ECG by increasing the dispersion of repolarization (Zhu and Clancy, 2007). In iPSC cells of a Timothy syndrome patient, a cyclin-dependent kinase inhibitor, roscovitine was found to shorten APD by partially recovering inactivation of the mutant channel (Yarotskyy et al., 2010; Yazawa et al., 2011). If Timothy syndrome/LQTS-8 is diagnosed, because of the high mortality, ICD implantation is the first choice. ICD is often supplemented with β-blockers, relying on the fact that they are generally effective in LQTS patients. Also, verapamil (Jacobs et al., 2006), mexiletine (Krause et al., 2011), and ranolazine (Shah et al., 2012) have been shown to shorten APD by affecting I_Ca,L_ and reducing the risk of arrhythmias.

LQTS-14–16 are newly described subtypes of LQT syndrome, caused by mutations in the genes coding the ubiquitous Ca^2+^ sensor and binder, calmodulin (CaM). Mutations in CALM1-encoding CaM1, CALM2-encoding CaM2, and CALM3-encoding CaM3 are responsible for producing LQTS-14, LQTS-15, and LQTS-16, respectively. Patients diagnosed with these conditions are usually young and have a high rate of cardiac arrest with severe QT prolongation (Gray and Behr, 2016). CaM is important in the inactivation of Na^+^ channels, Ca^2+^-dependent inactivation of I_Ca,L_ and also important in the trafficking, assembly, and gating of the I_Ks_ channel, KCNQ1 (Shamgar et al., 2006). Gene anomalies, affecting CaM, and therefore, Ca^2+^ binding and/or enhancing I_Ca,L_ can lead to severe APD prolongation. To date, over 20 mutations have been reported in the disease group of calmodulinopathies (Jensen et al., 2018; Wren et al., 2019) associated with LQTS, CPVT, and idiopathic VF. LQTS mutations, e.g. CaM-D130G, CaM-D96V, CaM-N98S, and CaM-F142L are all having impaired Ca^2+^ binding properties at the EF hand domains (Crotti et al., 2013). In CaM-D130G, CaM-D96V, and CaM-N98S mutations impaired CaM-dependent inhibition of RyR was reported, thereby increasing SR Ca^2+^ release due to an increased open state of RyR (Sondergaard et al., 2017; Jensen et al., 2018). Unexpectedly, an LQTS-associated CaM mutation, CaM-F142L did not diminish, but, increased the CaM-dependent RyR gating inhibition and caused faster RyR closing at high [Ca^2+^]_i_ (Sondergaard et al., 2017). The authors proposed that the mutation displayed both gain-of-function and loss-of-function properties. In the process of gain-of-function, F142L mutation increases the interactions between the C-domain of CaM and the CaM binding domain of RyR, therefore enhancing RyR inhibition. On the other hand, the loss-of-function effect impairs the ability of the C-domain of CaM to bind free Ca^2+^, i.e. decreases RyR inhibition. However, at high [Ca^2+^]_i_ C-domain of CaM saturates allowing the increased RyR inhibitory effect to be the dominant one (Sondergaard et al., 2017). One might assume an overlap between LQTS and CPVT as diminished inhibitory effect on RyR gating is generally associated with CPVT. In mutant guinea pig cells, it was shown that decreased inhibition of RyR gating with impaired CaM effect on the CaM-dependent inactivation of I_Ca,L_ (i.e. increased I_Ca,L_) may contribute to APD prolongation and that LQTS associated CaM mutations can lead to electrical alternans, a pathological feature of LQTS (Limpitikul et al., 2014).

Recently a novel mutation, LQTS-17 has been proposed, however, the nomenclature is still indistinct. Some reviews refer to LQTS-17 as a mutation in TRDN-encoded triadin, which has also been linked to CPVT-5 (Landstrom et al., 2017). However, Altmann et al., originally identified the autosomal recessive homozygous or compound heterozygous frameshift loss-of-function mutations in TRDN, proposed the term Triadin Knockout Syndrome (TKOS) or TRDN-mediated autosomal-recessive LQTS, rather than LQTS-17 (Altmann et al., 2015). As in the previous case, here is also the possibility of an overlap with CPVT, as QT prolongation and disease appearance at young age is accompanied by arrhythmias that occur during exercise. The possible cellular mechanism includes reduced negative feedback on I_Ca,L_ (i.e. increased I_Ca,L_), increased spontaneous Ca^2+^ release *via* RyR, and promotion of SR Ca^2+^ loading by NCX. It is not clear yet, whether the arrhythmogenic feature is mediated by DAD or EAD, but in a TRDN-null mice model, nifedipine aborted SR Ca^2+^ overload and spontaneous Ca^2+^ release (Chopra et al., 2009).

Although most of the LQTSs are inherited in an autosomal dominant form, there is a relatively rare, autosomal recessive inherited form, causing the Jervell and Lange-Nielsen syndrome (KCNQ1 or KCNE1, leading to reduced I_Ks_) (Splawski et al., 1997; Duggal et al., 1998). LQTS-related arrhythmias can be triggered by either slow or fast heart rate or by sinus pauses, therefore the relation between the LQTSs and the sinoatrial node is an interesting topic; for details, see the mini-review from Wilders and Verkerk (2018). For a detailed summary chart about LQTSs with the genetic loci, see a recent review of Landstrom et al. (Landstrom et al., 2017).

Pharmacological management of congenital LQTS starts with the administration of β-blockers, irrespective of the genotype (Moss et al., 2000). In one study, propranolol was shown to be the most effective β-blocker (Na^+^ channel blockade with limited effects on K^+^ channels) (Chockalingam et al., 2012). It should be noted that care is required with the use of β-blockers at low heart rate in LQTS-3 since bradycardia-dependent arrhythmias occur more often in these patients (El-Sherif et al., 2017). It was shown in LQTS-2 patients that besides β-blockers, application of mexiletine may also have positive effects (Kim et al., 2010; Ildarova et al., 2012). As an add-on therapy, in the case of LQTS-3 patients mexiletine (Schwartz et al., 1995), lidocaine, tocainide (Rosero et al., 1997), flecainide (Moss et al., 2005), phenytoin (Vukmir and Stein, 1991), or ranolazine (Moss et al., 2008) can be useful (Priori et al., 2013). In LQTS where mutations cause reduction in K^+^ currents, drugs that enhance K^+^ currents, nicorandil (Shimizu et al., 1998) or RPR26043 (Kang et al., 2005) were shown to be effective. ICD implantation is recommended for survivors of cardiac arrest or with recurrent syncope while on β-blocker (Priori et al., 2013). Left cardiac sympathetic denervation (LCSD) can also be performed on high-risk patients (arrhythmic events even in the presence of β-blocker/ICD). In addition to drugs or surgical procedures, lifestyle changes, such as avoidance of drugs that lengthen QT interval, identification and correlation of electrolyte abnormalities, avoidance of strenuous exercise (especially swimming in LQTS-1 patients) and abrupt loud noises (LTQS-2) are recommended for patients (Priori et al., 2013).

### Brugada Syndrome

Brugada syndrome (BrS) is characterized by ST elevation in V1–V3 ECG leads and is associated with elevated risk of polymorphic VT, VF, and sudden cardiac death (Brugada and Brugada, 1992). Two hypotheses have been proposed to describe the mechanism behind BrS and how ST segment elevation is linked to VT/VF. (Ringer, 1883) In the repolarization hypothesis, the loss of spike-and-dome AP morphology (heterogenous shortening of AP due to predominance of I_to_ over I_Na_ and I_Ca,L_) is suggested in the epicardium of the right ventricular outflow tract, causing an enhanced transmural dispersion of repolarization, i.e. ST elevation (Yan and Antzelevitch, 1999). The arrhythmogenic mechanism is delivered by phase-2 reentry, when the produced extrasystole can occur on the preceding T wave (R-on-T phenomenon), finally initiating VT/VF. (Bers, 2002) The depolarization theory proposes a slowed conduction and delayed activation mechanism in the right ventricular outflow tract as a substrate for reentry (Meregalli et al., 2005).

To date, 23 gene (gain-of-function and also loss-of-function) mutations have been described generating BrS-1–BrS-23 (Gray and Behr, 2016). The most common subtype is BrS-1, mutation affects the SCN5A-encoded α-subunit of the Na^+^ channel (Na_v_1.5) and is accountable for about one third of all BrS (Antzelevitch et al., 2005). Genes, governing Ca^2+^-signaling molecules are also affected in BrS and causing 10–15% of cases (Burashnikov et al., 2010) (**Table 1**). Loss-of-function mutation of the CACNA1C-encoded α_1C_-subunit (Ca_v_1.2α1; BrS-3), the CACNB2-encoded β_2_-subunit (Ca_v_β2; BrS-4), and the CACNA2D1-encoded α_2_δ_1_-subunit (Ca_v_α2δ1; BrS-11) of the L-type Ca^2+^ channel (governing I_Ca,L_) have been described with a concomitant reduction of I_Ca,L_ (Antzelevitch et al., 2007). Patients harboring these Ca^2+^ related mutations showed BrS like ECG but with shorter than normal QT intervals. Recently, a new Ca^2+^-related mutation has been linked to BrS, accounting for about 6% of the cases. Mutation of the TRPM4-encoded Ca^2+^ activated non-selective cation channel transient receptor potential melastatin 4 (TRPM4; BrS-15) can either be gain-of-function or loss-of-function (Liu et al., 2013). TRPM4-mediated current increases APD in atrial muscle and isolated myocytes (Simard et al., 2013), possibly by promoting the plateau (as it is more likely to activate when Ca^2+^ is elevated). Therefore, TRPM4 mutation may change the AP dome and be arrhythmogenic. TRMP4 may also slow down conduction by altering the availability of Na^+^ channels (Liu et al., 2013).

There have been pharmacological attempts to manage BrS (isoproterenol, quinidine, procainamide, propafenone, pilsicainide, flecainide), some of them were effective in preventing recurrent episodes of VF or electrical storms, but did not reduce the overall risk of VF (Brugada et al., 2000; Shimizu et al., 2000; Morita et al., 2003; Belhassen et al., 2004; Ohgo et al., 2007). Guidelines are also recommending lifestyle changes (omit drugs that aggravate ST elevation, avoid alcohol and immediate treatment if fevered) and implantation of ICD (Priori et al., 2013).

### Short QT Syndrome

Short QT syndrome (SQTS) is a rare inherited syndrome characterized by QT intervals essentially shorter than 360 ms and by an increased incidence of VT/VF mainly in youngsters (Bjerregaard et al., 2010). There are eight different gene mutations, of which three affect I_Ca,L_ (**Table 1**). Loss-of-function mutation of the CACNA1C-encoded α_1C_-subunit (Ca_v_1.2α1; SQTS-4), the CACNB2-encoded β_2_-subunit (Ca_v_β2; SQTS-5), and the CACNA2D1-encoded α_2_δ_1_-subunit (Ca_v_α2δ1; SQTS-6) of the L-type Ca^2+^ channel, similar to the BrS-3, BrS-4, and BrS-11 phenotype. These mutations decrease I_Ca,L_ (alter current density and activation/inactivation kinetics), cause heterogenous shortening of APD and QT interval, therefore increases dispersion of repolarization (Antzelevitch et al., 2007). Transmural dispersion of repolarization (shortening effect is more pronounced in the epicardium compared to endocardium and midmyocardium) finally serves as a substrate for reentry. These mutations combined with the mutation of SCN5A-encoded α-subunit of the Na^+^ channel (Na_v_1.5) causes an overlapping phenotype of SQTS and BrS.

### Early Repolarization Syndrome and Idiopathic Ventricular Fibrillation

Early repolarization syndrome (ERS) is characterized by J-point and ST segment elevation in two or more contiguous leads on ECG (Boineau, 2007). The early repolarization pattern (in the inferior and/or lateral precordial leads) had been considered harmless, but it has recently been associated with idiopathic ventricular fibrillation (IVF) (Rosso et al., 2008). ERS now is diagnosed in IVF survival patients, without other causes of cardiac arrest (channelopathies; structural or non-structural heart diseases, e.g. BrS; metabolic; toxicological; respiratory; and infectious) (Haissaguerre et al., 2008). Seven gene mutations were shown, to date, including loss-of-function mutations of CACNA1C, CACNAB2, and CACNA2D1, as seen in BrS or SQTS (**Table 1**). L-type channel mutations account for 16% of cases (Burashnikov et al., 2010). CaM-F90L mutation was proposed to be linked to IVF phenotype, where the authors speculated that CaM mutations could be arrhythmogenic by altering Ca^2+^ binding and/or binding of target proteins, thus generating a rather insensitive CaM and that the gene anomaly is more pronounced in the Purkinje system (Marsman et al., 2014). Recently, a novel single point mutation in RyR2 (RyR2-H29D) has been linked to IVF phenotype (Cheung et al., 2015). RyR2-H29D mutation was shown to be associated with short-coupled premature ventricular contractions, initiating polymorphic VT. This mutation caused diastolic Ca^2+^ leak at rest by higher open probability and higher frequency of opening of RyR at low diastolic Ca^2+^ levels in a non-PKA phosphorylated state, unlike the typical CPVT-related RyR mutations. Therefore, RyR dysfunction caused by RyR2-H29D mutation may play a role in short-coupled polymorphic VT.

J-point elevation associated malignant arrhythmias have recently been proposed with a new classification, as J-wave syndrome (Antzelevitch and Yan, 2010).

## Acquired Syndromes

### Acquired Long QT Syndrome

In addition to the congenital form, LQTS can also be acquired. The prevalence of acquired LQTS is greater than that of congenital forms (El-Sherif et al., 2019). It is generally caused by adverse, unwanted drug effects and/or electrolyte abnormalities and may predispose to the prolongation of the APD/QT interval, increase in dispersion of refractoriness and to a higher risk for generating EADs, being the substrates for VTs, especially for torsade de pointes VT (El-Sherif and Turitto, 1999).

The above mentioned effects are often seen for the hERG-encoded (human ether-à-go-go-related gene or KCNH2) K_v_11.1 channel, responsible for I_Kr_ while effects on enhanced I_Na,L_ has also been reported (Yang et al., 2014). The role of dispersion of repolarization in generating tachyarrhythmias (and the role as a preclinical proarrhythmia marker) is further supported by a series of experiments, where DL-sotalol and amiodarone were compared (Milberg et al., 2004). It was shown, that both hERG-blockers increased QT interval, however only DL-sotalol increased transmural dispersion of refractoriness, EADs and torsade de pointes (and caused triangulation of the AP), while amiodarone caused phase-2 prolongation of the AP without triangulation, which is otherwise considered proarrhythmic.

Several other causes of acquired LQTS have been described, including electrolyte disorders (El-Sherif and Turitto, 2011), such as hypokalemia, hypomagnesemia or hypocalcemia, hypothyroidism, hypothermia, but also antidepressant and antipsychotic treatments (Sicouri and Antzelevitch, 2018), female gender, and autoimmune and inflammatory diseases (Lazzerini et al., 2015; Boutjdir et al., 2016). Hypocalcemia causes QT prolongation *via* phase-2 prolongation of AP (Eryol et al., 2003), also longer and late Ca^2+^ influx (due to reduced Ca^2+^-dependent inactivation of I_Ca,L_) can favor the formation of EADs.

### Atrial Fibrillation

The most prevalent cardiac arrhythmia is atrial fibrillation (AF) and this can be classified as paroxysmal (spontaneously self-terminates into sinus rhythm in less than 7 days), persistent (lasts for more than 7 days), long-lasting persistent (AF lasts for more than a year) or permanent AF (without active rhythm control) (Kirchhof et al., 2016). AF is multifactorial. Basic arrhythmogenic mechanisms include Ca^2+^ handling defects such as triggered activity (DAD, late-phase 3 EAD), conduction block (reentry), and Ca^2+^-driven cardiac alternans and altered Ca^2+^ buffering (Nattel and Dobrev, 2016). DAD-mediated triggered arrhythmias are underlined by Ca^2+^ handling instability in AF, namely RyR dysfunction (increased phosphorylation and open probability), increased SERCA function, increased diastolic SR Ca^2+^ leak and spontaneous SR Ca^2+^ release, increase in Ca^2+^ sparks and waves, enhanced CaMKII function (with subsequent RyR hyperphosphorylation), or reduced I_Ca,L_ (Sood et al., 2008; Neef et al., 2010; Shan et al., 2012; Voigt et al., 2012). Involvement of late-phase 3 EAD has also been shown (Burashnikov and Antzelevitch, 2006). As in most of the AF models APD is abbreviated, this observation can be somewhat surprising, since EADs generally occur at a prolonged APD. However, as we previously described, late-phase 3 EADs occur at shorter APD and at elevated Ca^2+^ loading conditions (such as rapid atrial pacing). These ectopic activities can serve as a trigger for reentry which is considered to be the main arrhythmogenic mechanism in AF. Also, I_Ca,L_ reduction in AF causes APD shortening and promotes reentrant activity (Heijman et al., 2014). Reduction of I_Ca,L_ might be governed by reduction of protein and mRNA levels of the channel (alpha subunit) after rapid pacing. This transcriptional downregulation of Ca^2+^ channel has been proposed to be mediated by activation of calcineurin by Ca^2+^/CaM, which in turn, regulates nuclear translocation of NFAT (Qi et al., 2008).

A novel, interesting theory has been proposed, namely, Ca^2+^ signaling silencing, as an antiarrhythmic adaptive mechanism in AF (Greiser et al., 2014). The key observation was, that sustained high atrial pacing may not lead to Ca^2+^ instability, suggesting a role of accompanying cardiovascular diseases (e.g. HF) rather than “lone AF” itself in those cases when unstable Ca^2+^ signaling occurs in AF. Ca^2+^ signaling silencing process includes the failure of centripetal intracellular Ca^2+^ signal propagation (also unchanged level of Ca^2+^ sparks and decreased amplitude of the systolic Ca^2+^ transient), remodeling of the RyR complex (reduced protein expression and CaMKII-mediated phosphorylation), and lower Na^+^ concentration (consequential reduction in Ca^2+^ load) (Greiser, 2017). The decreased propagation was associated with an increase of cytoplasmic buffer power possibly due to increased Ca^2+^ sensitivity of myofilaments resulting from decreased phosphorylation of troponin I (Greiser et al., 2014). The authors concluded that the Ca^2+^ signaling phenotype in AF patients is a net result of factors that stabilize (i.e. Ca^2+^ signaling silencing) or destabilize it (arrhythmogenic Ca^2+^ instability). Therefore, future therapeutic approaches should identify the substrate (arrhythmia enhancing abnormalities or arrhythmia suppressing Ca^2+^ signaling silencing) and tailor therapies for individual AF patients (Kirchhof et al., 2016; Schotten et al., 2016; Greiser, 2017).

For an excess review about the role of Ca^2+^ in the pathophsiology of AF see the review of Denham et al. (2018).

## Conclusions

In summary, we have reviewed the roles of Ca^2+^ in cardiac E-C-coupling focusing on those defects which lead to cardiac arrhythmias in inherited and acquired syndromes. In the last few decades there have been great advances in the understanding of these arrhythmias, however, there is still a need for more work investigating the physiology and pathophysiology of Ca^2+^ related events. Designing drugs to treat a specific disease type has never been simple; it is enough to think of the early disappointing attempts to block the Na^+^ or K^+^ channels (CAST and SWORD trials, respectively). Multiple characteristics of novel therapeutic approaches have to be determined and to be considered as a complex, systems problem.

Along with the generally used β-blockers, newly developed selective drugs without proarrhythmic side effects are necessary. While implantable cardiac defibrillators provide longer life expectancy, they cannot prevent the onset of cardiac events. An additional helpful tool would be reliable and effective risk stratification and clinical guidance for all of the syndromes discussed. It should not be overlooked that in the future other genetic mutations may be discovered requiring novel biological therapies. Because of the diversity of inherited and acquired mutations individually tailored therapeutic approaches (gene-specific or mutation-specific pharmacological and/or gene therapy) will be required.

To gain a better understanding of the role of Ca^2+^ in the cardiac arrhythmias data from basic science should meet the clinical practice; translational aspects must be key in all fields of science.

## Author Contributions

KK conceived the review and drafted the manuscript. KK, RV, BH, TB, PN, and DE revised the manuscript critically for important intellectual content. DE contributed to the critical review of the literature, editing of the manuscript text and review of the figures. All authors approved the final version of the manuscript submitted.

## Funding

This work was funded by the National Research Development and Innovation Office (NKFIH-K115397). Further support was obtained from GINOP-2.3.2.-15-2016-00040 and EFOP-3.6.2-16-2017-00006 projects, which are co-financed by the European Union and the European Regional Development Fund. The research was financed by the Thematic Excellence Programme of the Ministry for Innovation and Technology in Hungary (ED_18-1-2019-0028), within the framework of the Space Sciences thematic program of the University of Debrecen. This work was supported by the British Heart Foundation Chair Award (grant number: CH/200004/12801).

## Conflict of Interest

The authors declare that the research was conducted in the absence of any commercial or financial relationships that could be construed as a potential conflict of interest.

## Abbreviations

AF, atrial fibrillation; AP, action potential; APD, action potential duration; AV, atrioventricular; BrS, Brugada syndrome; CaM, calmodulin; CaMKII, Ca^2+^/calmodulin-dependent protein kinase II; CICR, Ca^2+^-induced Ca^2+^ release; CPVT, catecholaminergic polymorphic ventricular tachycardia; CSQ2, calsequestrin 2; DAD, delayed afterdepolarization; EAD, early afterdepolarization; EC, excitation-contraction coupling; ERS, early repolarization syndrome; HF, heart failure; ICD, implantable cardiac defibrillator; IVF, idiopathic ventricular fibrillation; LQTS, long QT syndrome; NCX, sodium-calcium exchange; NFAT, nuclear factor of activated T-cells; PKA, protein kinase A; PLN, phospholamban; PMCA, plasma membrane Ca^2+^-ATPase; PVC, premature ventricular contraction; RSV, relative short term beat-to-beat variability of action potential duration; RyR, ryanodine receptor; SA, sinoatrial; SERCA, sarco/endoplasmic reticulum Ca^2+^-ATPase; SOCE, store overload-induced Ca^2+^ entry; SOICR, store overload-induced Ca^2+^ release; SQTS, short QT syndrome; SR, sarcoplasmic reticulum; SV, short term beat-to-beat variability of action potential duration; VF, ventricular fibrillation; VT, ventricular tachycardia.
